# Single-cell RNA sequencing reveals anterograde trans-synaptic degeneration and exacerbated synaptic remodeling in myopia

**DOI:** 10.1038/s12276-025-01489-y

**Published:** 2025-07-03

**Authors:** Ruixue Zhang, Yunxiao Xie, Miao Zhang, Zhiheng Li, Lijie Guo, Huixia Wei, Xiaomeng Li, Jizhao Xin, Zhaohui Yang, Ying Wen, Jiawen Hao, Xianglin Li, Xuewei Yin, Wenjun Jiang, Hongsheng Bi, Dadong Guo

**Affiliations:** 1 Shandong University of Traditional Chinese Medicine, Jinan, China; 2 Affiliated Eye Hospital of Shandong University of Traditional Chinese Medicine, Jinan, China; 3 Department of Pathology, School of Basic Medical Science, Capital Medical University, Beijing, China; 4 Guangzhou laboratory, Guangzhou Medical University, Guangzhou, China; 5 School of Ophthalmology and Optometry, Wenzhou Medical University, Wenzhou, China; 6 School of Medical Imaging, Binzhou Medical University, Yantai, China; 7 Medical College of Optometry and Ophthalmology, Shandong University of Traditional Chinese Medicine, Key Laboratory of Traditional Chinese Medicine Classical Theory, Ministry of Education, Jinan, China; 8 Shandong Provincial Key Laboratory of Integrated Traditional Chinese and Western Medicine for Prevention and Therapy of Ocular Diseases, Jinan, China; 9 Shandong Academy of Eye Disease Prevention and Therapy, Jinan, China

**Keywords:** Experimental models of disease, Diagnostic markers

## Abstract

Myopia is a serious public health issue worldwide. Damage to retinal ganglion cells (RGCs) in the retina induces degeneration of the visual cortex, which is known as anterograde trans-synaptic degeneration (TSD). However, the role of TSD in myopia is still unknown. Here single-cell RNA-sequencing revealed the activation of RGC apoptotic signals in the retinal ganglion and the remodeling of synapses in the visual cortex in myopia. The thickness of the retinal nerve fiber layer was negatively correlated with the degree of damage to the visual cortex and damage to neurons in the visual pathway and to the synaptic structure and function of the visual cortex indicated the occurrence of anterograde TSD in the visual pathway. The knockdown of Fos, which inhibited retinal neuronal apoptosis, suppressed TSD, indicating that myopia can aggravate RGC apoptosis, induce anterograde TSD and thus aggravate synaptic remodeling. Our findings provide a new experimental basis for understanding the pathogenesis of myopia.

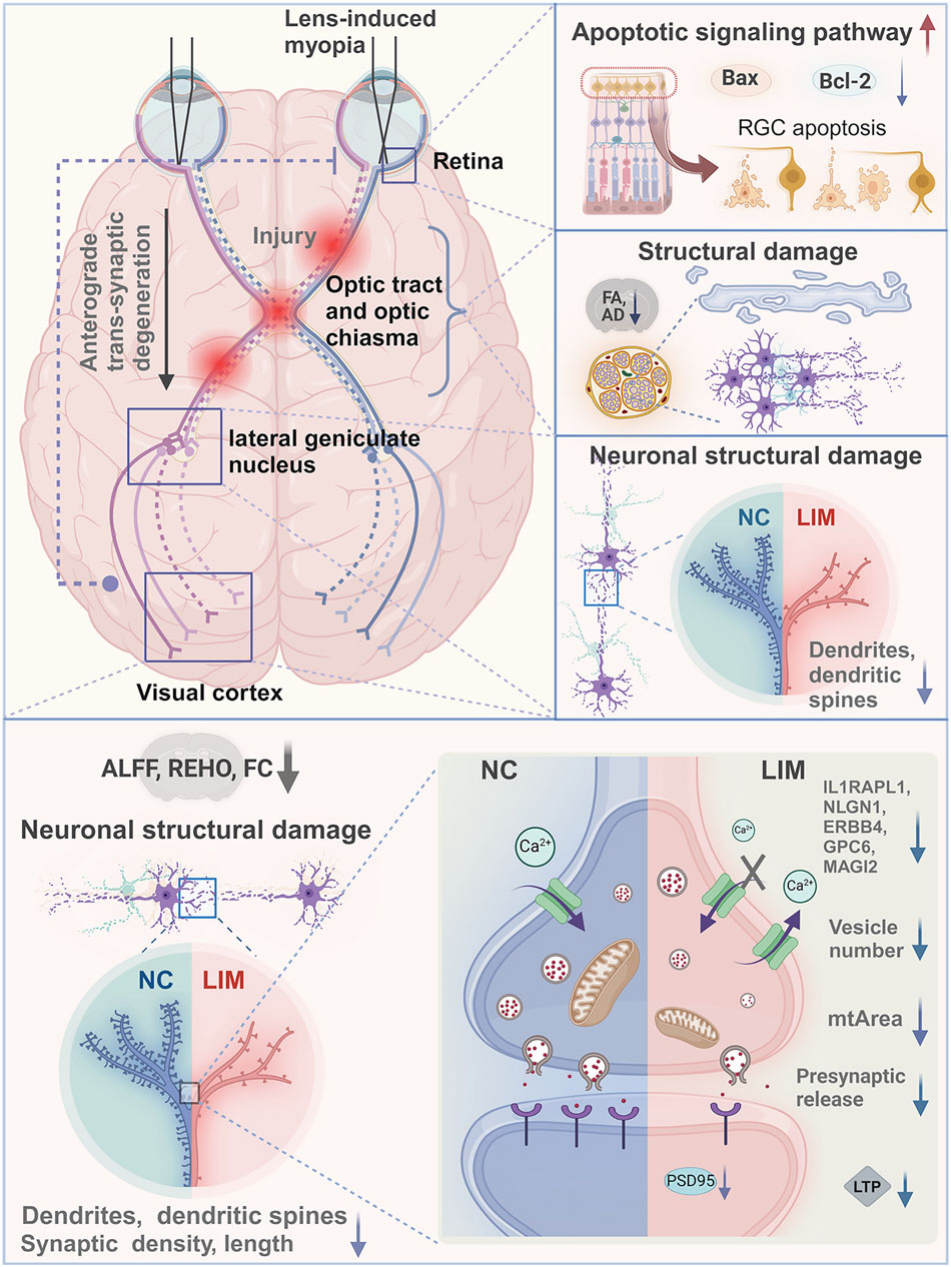

## Myopia linked to retinal ganglion cell apoptosis and damage

Myopia, or nearsightedness, is a common eye condition affecting many people worldwide. Here researchers explored whether a process called anterograde trans-synaptic degeneration (TSD) is involved in myopia. TSD happens when damage in one part of the visual system leads to degeneration in another part. In this study, guinea pigs were used to model myopia by fitting them with special lenses. The researchers examined changes in the retina and visual cortex using various techniques such as single-cell RNA-sequencing and imaging. They found that myopia caused retinal ganglion cells to undergo apoptosis, which led to changes in the visual cortex. The results suggest that retinal ganglion cell apoptosis contributes to TSD, affecting the visual pathway and potentially worsening myopia. The study highlights the importance of understanding these mechanisms for developing better treatments for myopia.

This summary was initially drafted using artificial intelligence, then revised and fact-checked by the author.

## Introduction

Myopia is one of the most common eye diseases in children and adolescents worldwide and is a major public health problem. The number of people with myopia is increasing rapidly: one study predicted that by 2050, 49.8% of the world’s population will suffer from myopia and that 9.8% will have high myopia^1^. Notably, for each additional diopter (D) of myopia, the risk of myopia-induced maculopathy, open-angle glaucoma, posterior subcapsular cataract and retinal detachment increases by 58%, 20%, 21% and 30%, respectively^2^. However, the mechanism of myopia is still not fully understood. Damage to the retinal ganglion leads to degeneration of the visual cortex, a process known as anterograde trans-synaptic degeneration (TSD). Retinal degenerative diseases, such as glaucoma and myelin oligodendrocyte glycoprotein antibody-associated disease, can cause TSD in the visual pathway, resulting in damage to the visual cortex^3,4^. However, whether TSD is also involved in myopia remains unclear. As apoptosis occurs in the retina in myopia^5,6^, we hypothesized that apoptosis in the retina in myopia may induce anterograde TSD of the visual pathway, leading to degeneration of the visual cortex.

The visual pathway refers to the entire visual information transmission pathway from the retina, which receives light signals, to the cerebral cortex, which forms vision, including the retina, optic nerve, optic chiasma, optic tract, lateral geniculate nucleus (LGN), optic radiation and visual cortex. Retinal ganglion cells (RGCs), whose spike activity is responsible for all the information the brain receives from the visual world, are the only projection neurons in the retina and have been extensively studied for their diversity, connectivity, signaling and development^7^.

Approximately 80% of the visual information that the retina projects to the visual center via the optic nerve is projected to the LGN. The main projection pathway of the LGN is to project encoded visual information to the primary visual cortex region V1, which is the main source of receiving neural projections in the primary visual cortex^4^. Previous studies have shown that apoptotic degeneration of the retina can lead to atrophy of the visual cortex, resulting in anterograde TSD of the visual pathway^8,9^. One myopia-related study revealed that myopia can change the thickness of the RGC layer^5^. Moreover, in form-deprivation myopia (FDM) guinea pigs, RGCs are unevenly distributed, accompanied by a decrease in the number of RGCs and an increase in apoptosis^6^. Studies on the correlation between myopia and changes in the visual cortex have shown that high myopia significantly disturbs functional connectivity (FC) between the primary visual cortex (V1) and several other brain regions, including the anterior part of the left cingulate gyrus, the left supraparietal gyrus, the left calcarine cortex and the right lingual gyrus^10^. In addition, the percentage change in blood oxygen level-dependent (BOLD) signal intensity in the visual cortex of myopic patients is significantly lower than that in normal subjects^11^. Nevertheless, the relationship between the retina and the visual cortex in myopia has not been investigated.

Single-cell RNA-sequencing (scRNA-seq) can provide unique physiological insights into the relationship between the optic cortex and myopia. However, the cell type and regional specificity in the visual cortex of myopic guinea pigs have not yet been investigated. Although there have been some reports on the expression levels of factors in the optic cortex of patients with myopia^12^, there has been no in-depth study of the retina and optic cortex in myopic eyes at the single-cell level. To better understand the role of the retinal ganglion‒optic cortex in the pathophysiology of myopia, it is necessary to conduct cell type-specific studies of the myopic retina and the optic cortex of the brain.

In this study, we first investigated the role of retinal and cortical heterogeneity in myopia progression via scRNA-seq. We used functional magnetic resonance imaging (fMRI), patch clamp, Golgi staining, electron microscopy, immunohistochemistry, immunofluorescence and other techniques to study the synaptic transmission efficiency and structure of visual cortex neurons. Our study confirms the occurrence of anterograde TSD that leads to inhibition of synaptic transmission in the visual cortex in myopia.

## Materials and methods

### Animals

Guinea pigs (*Cavia porcellus*, British shorthair, tricolor strain) with average weights between 130 and 150 g were provided by Jinan Jinfeng Experimental Animal Company. Before the study, guinea pigs with cataracts or corneal diseases were excluded, and healthy animals were randomly divided into the normal control (NC) group, lens-induced myopia (LIM) group, AAV-Fos group and AAV-hSyn-tettoxic group. Guinea pigs in the NC group received no treatment, whereas those in the LIM group were fitted with −6.0D lenses on the right eye to induce myopia. On the first day of myopia induction at enrollment, the Guinea pigs in the AAV-FOS group wore a −6.0D lens to induce myopia and received an intravitreal injection of 6 μl (ref. ^13^) AAV5-shRNA-Fos (CCTGTCTAGTTCATTCTAT) with a viral titer of 1.14 × 10^12^ vector genomes per ml (VG/ml) (OBiO) to knock down the retinal Fos gene. Moreover, animals in the AAV-hSyn-tettoxlc group received an intravitreal injection of 6 μl of AAV-hSyn-tettoxlc (rAAV-hSyn-tettoxlc-EGFP-WPRE-hGH polyA, BrainVAT) at a viral titer of 1.14 × 10^12^ VG/ml to inhibit synaptic transmission^14^ (*n* = 47).

### Myopia diopter and axial length

After 4 and 6 weeks of myopia induction, ophthalmic type A ultrasonography (Cinescan, Quantel Medical) was used to measure the axial length, and each eye was measured at least ten times. The anterior chamber propagation velocity was 1557 m/s, the lens propagation velocity was 1723 m/s and the vitreous propagation velocity was 1540 m/s. The average of ten measurements was the final reading.

### ScRNA-seq

#### Animal tissues

The tissues from guinea pigs in the NC and LIM groups (4 weeks) were assayed with scRNA-seq (for the retina and visual cortex) and single-nucleus RNA-sequencing (snRNA-seq, visual cortex only). In the present study, the left visual cortex tissues of guinea pigs were dissected into 3 mm^3^ fragments and used for snRNA-seq and scRNA-seq.

#### Quality control

Newly generated sequencing data were aligned to the Cavpor3.0 guinea pig reference genome and quantified using Cell Ranger (version 3.0, 10x Genomics Inc.). The preliminary filtered data generated from Cell Ranger were then used for downstream filtering and analyses. The quality of the cells was then assessed on the basis of two metrics: (1) the number of genes detected per cell and (2) the proportion of mitochondrial gene counts per cell. Specifically, cells were filtered out if they had fewer than 200 detected genes, unique molecular identifiers (UMIs) exceeding 40,000 (duplets) or a mitochondrial UMI count percentage greater than 10%.

#### Removal of the batch effect

We combined the scRNA-seq and snRNA-seq data of the same tissue (for the visual cortex). We used the RunHarmony function of the Harmony package (version 1.0.1) to eliminate batch effects of different samples in the principal component space with default parameters. In the present study, the top 20 harmony components were processed using the RunUMAP function to embed and visualize the cells in a two-dimensional map.

#### Cell annotation

To annotate the cell clusters, we identified the markers of each cluster via the FindAllMarkers function in the Seurat package (version 4.3.0.1) with default parameters. We considered only those genes that were expressed in at least 10% of the cells in any tested cluster with a |log2 fold change| >1.5 and a Bonferroni-corrected *P* value <0.05 as markers. The cell clusters were then annotated according to the markers combined with curated known cell markers from the literature^15^. Clusters expressing the same cell markers were merged.

#### DEGs between LIM and NC animals

To filter differentially expressed genes (DEGs) in LIM animals compared with NC animals, the genes in at least 25% of the cells of one tested cell type with |average_log2 fold change| >0.25 and a Bonferroni-corrected *P* value <0.01 were regarded as DEGs.

#### Enrichment analysis

The enriched biological functions of DEGs in each cell type were analyzed via the enrich GO and enrich KEGG functions of the clusterProfiler^16^ package (version 4.8.3) in R. The enriched Gene Ontology (GO) terms and Kyoto Encyclopedia of Genes and Genomes (KEGG) pathways were filtered with the *P* value adjusted via the Benjamini‒Hochberg method (adjusted *P* value <0.05).

### FVEP

The flash visual evoked potential (FVEP) assay was performed at 4 and 6 weeks using the OPTOPROBE Ophthalmic Imaging System (Optoprobe). Guinea pigs were adapted to dark conditions and underwent visual electrophysiological examinations in a red-light environment. The animals were then positioned on the operating table and electrodes were placed appropriately.

The electrode setup for the FVEP was as follows: the positive electrode was positioned in the subcutaneous layer corresponding to the occipital lobe of the brain, the negative electrode was placed on the cheek and the grounding was located in the subcutaneous layer at the tail. The flashing light was the stimulating light, the flashing frequency was 1 Hz and there were 60 flashes for each detection. The P1 and P2 wave amplitude and latency values were recorded at the end of the test.

### MRI data acquisition

MRI scanning was performed using a Bruker 7.0T small animal MR instrument (Bruker BioSpec USR 70/20, Paravision 6.0.1).

The T2WI (TurboRARE sequence) scan parameters were as follows: total retinal (TR) 3,790 ms, echo time (TE) 35 ms, average 4, slice 29, slice thickness 1 mm, image size 256 × 256, field of view 35 × 35 mm^2^; diffusion tensor imaging (DTI) (echo planar imaging (EPI) sequence) scan parameters: TR 2,000 ms, TE 34 ms, average 4, repetition 1, b value 1,000 s/mm^2^ taking 30 different directions, slice 29, slice thickness 1 mm, image size 96 × 96 and field of view 35 × 35 mm^2^. The DTI (EPI sequence) scan parameters were as follows: TR 2,000 ms, TE 34 ms, average 4, repetition 1, b value 1,000 s/mm^2^ in 30 different orientations, slice 29, slice thickness 1 mm, image size 96 × 96 and field of view 35 × 35 mm^2^. The BOLD (SE-EPI sequence) scan parameters were as follows: TR 2,000 ms, TE 19 ms, average 1, repetition 180, slice gap 0 mm, slice 29, slice thickness 1 mm, image size 96 × 96 and field of view 35 × 35 mm^2^.

#### BOLD image processing

The first ten points were discarded from the MRI signal to allow the guinea pigs to adapt to the environment. BOLD data were acquired using SPM12 (ref. ^17^) and the Resting-State fMRI Data Analysis Toolkit (version 2.8; http://www.restfmri.net). Image processing included the following steps: (1) slice timing, (2) head motion correction and realignment, (3) spatial normalization to standard guinea pig brain stereotaxic coordinates, (4) nuisance covariate regression, (5) temporal bandpass filtering (0.01–0.08 Hz), (6) smoothing with a 6 mm full-width at half maximum isotropic Gaussian kernel and (7) extracting the mean amplitude of low-frequency fluctuation (ALFF) (mALFF = voxel ALFF/mean of whole-brain ALFF) and smoothed-mean regional homogeneity (ReHo) (smReHo = voxel ReHo/mean of whole-brain ReHo) values of altered guinea pig brain clusters.

#### DTI data

The acquired images were transformed from the original format of DICOM to NIFTI, and voxels were resized by a factor of 10 to make the guinea pig brain volume similar to that of the human brain and match the requirements of most software. DTI maps of guinea pig heads were acquired using DSI Studio (http://www.dsi-studio.labsolver.org), FSL (http://www.fsl.fmrib.ox.ac.uk) and Mricron (http://www.people.cas.sc.edu/rorden/mricron).

#### FC data

We used Analysis of Functional Neuroimages (AFNI) analysis software to process the fMRI images and perform linear regression analysis. To eliminate signal interference, we extracted the bilateral caudate nuclei from standard template images as regions of interest, obtained the average time series values of the bilateral caudate nuclei and performed functional connectivity (FC) analysis with voxels from various brain regions.

### sEPSC and sIPSC

Whole-cell patch-clamp recording in voltage-clamp mode was used to record the postsynaptic current of visual cortex neurons. When negative pressure was applied to break the cell membrane and reach a stable state (leak <−30 pA, Ra <20 MΩ), the membrane potential was held at −70 mV, and the spontaneous excitatory postsynaptic current (sEPSC) and spontaneous inhibitory postsynaptic current (sIPSC) of the visual cortex of the brain were recorded.

### Acute slice preparation and LTP recording

The brain tissue of the guinea pigs was removed from the precooled slicing solution, and the cerebral hemispheres were trimmed and quickly transferred to the slicing tank, which was filled with precooled slicing solution and continuously filled with a 95% O_2_ + 5% CO_2_ gas mixture. The slicing solution inside the tank was maintained in the state of an ice‒water mixture. The parameters of the vibrating slicer were adjusted (400 μm) to section the whole brain tissue in the coronal position and the sliced visual cortex slices were placed in the incubation tank with the recording solution for incubation, during which the visual cortex structures were not damaged to prevent damage to the neuronal cells. The incubator was continuously filled with 95% O_2_ + 5% CO_2_. After 30 min of incubation, the recording fluid was replaced, and the temperature was controlled to 24 °C for 1 h before electrophysiological testing was performed on the machine. The slicing fluid and the artificial cerebrospinal fluid were continuously ventilated to maintain fluid homeostasis during testing.

After the brain slices were incubated, they were transferred to a perfusion tank to secure them at the center of the field of view, ensuring that the entire slice was continuously perfused with the recording fluid. The resistance of the recording electrode was checked and was used only when it measured between 3 and 5 MΩ. Under the microscope, the recording electrode was positioned in layers II/III of the visual cortex, whereas the stimulating electrode was placed in layer IV. The reference electrode was grounded, and electrical stimulation was applied to induce field excitatory postsynaptic potentials (fEPSPs).

After the maximum power of the stimulation was determined, it was adjusted to 50% of this maximum to induce a baseline fEPSP. Recording of the baseline fEPSP began once stabilization was achieved and continued for 20 min. Following the baseline fEPSP recording, long-term potentiation (LTP) was induced by administering two trains of high-frequency stimulation, each consisting of 100 pulses at 100 Hz spaced 20 s apart through the stimulating electrodes. LTP was measured by assessing the change in fEPSP amplitude.

### Ca^2+^ flux measurements

Noninvasive micro-test technology (NMT) (Younger USA LLC) was used to measure the Ca^2+^ fluxes in the retinal and visual cortex tissues. Before the measurements, a calcium ion-selective microelectrode was prepared as described previously^18^. The retina and visual cortex tissues were placed in a Petri dish containing 5 ml of artificial cerebrospinal fluid and a lid mesh was used to compress the tissue, securing the brain slice in place.

Using a three-dimensional propulsion device, the test electrode filled with LIX and high-calcium electrolyte liquid was quickly positioned on the edge tissue of the brain slice, the electrode was then accurately moved to approximately 50 μm above the tissue, and the position was automatically and reciprocally changed in the *z*-axis direction at a fixed distance of 30 μm. To measure the changes in Ca^2+^ concentration occurring outside the tissue membrane, the electrode movement frequency, or sampling frequency, was set at 0.3 Hz. After recording for 5 min, the raw data microvolt differences (ΔμV) were imported and converted into the Ca^2+^ flux using JCal version 3.3.

### TEM

The visual cortex was promptly excised and rinsed in 0.1 mol/l phosphate buffer (pH 7.4), followed by the immediate addition of a 3% glutaraldehyde fixing solution (pH 7.4). The sample blocks were then trimmed to 1 mm × 1 mm × 3 mm, rinsed again and fixed with 1% osmium tetroxide. After rinsing and dehydration, the samples were soaked and embedded in Epon 812, adhering to conventional transmission electron microscopy (TEM) preparation methods. Once the semi-thin sections were prepared, ultrathin sections measuring 70–100 nm were obtained using an LKB-V ultrathin microtome. The sections were observed via electron staining with lead citrate and uranium acetate with a JEOL-1200E transmission electron microscope, and the images were recorded with a MORADA-G2 system.

### RT‒qPCR

Six retinal tissues from each group were collected for molecular detection by quantitative PCR with reverse transcription (RT‒qPCR) or western blotting (1:1). Total RNA was extracted (Invitrogen, 15596026) and stored in cryogenic tubes (NEST Biotechnology) at −80 °C. The RNA purity and concentration were measured by an ultraviolet spectrophotometer (K5600; Beijing Kaiao Technology Development Co.). HiScript II Q RT SuperMix for qPCR (+gDNA wiper) (Shandong Sparkjade Biotech. Co., Ltd.) was used for reverse transcription to obtain cDNA, and reverse transcription of the total RNA to cDNA was performed (Shandong Sparkjade Biotech. Co., Ltd.), followed by RT‒qPCR using the SYBR Green I Master Kit (Roche, 4707516001). The primer sequences are listed in Table 1. The qPCR conditions were as follows: 94 °C for 5 s, 1 cycle; 94 °C for 5 s, 54 °C for 15 s and 72 °C for 10 s for 45 cycles, and the specificity of the RT‒qPCR product was confirmed by melting curve analysis. The target gene levels were normalized to those of the housekeeping gene β-actin, and the results were analyzed via the 2^−ΔΔCt^ method.

**Table 1:** The primer sequence of the target genes.

Gene	Primer sequence
ERBB4	F: 5′-CCCACCCGAGAAATECCTGACTTA-3′
	R: 5′-GGCGCTTCTGGGATGGTGCTGGTT-3′
MAGI2	F: 5′-AACGAGAACGGAGTGGTGGTGACG-3′
	R: 5′-CCTCGGGETTGCTGGACTTGGTGT-3′
IL1RAPL1	F: 5′-GAGGGCTTGGTGCTATTCTTTTAC-3′
	R: 5′-TTCTTCTTCCCCAGTCTCTTGATT-3′
NLGN1	F: 5′-ACECATGCCCASGAAGAGGAAATC-3′
	R: 5′-GAATGGGGGTGGGGCAGAGTA T-3′
PLP1	F: 5′-GGGCCTGAGCGCAACGGTAAC-3′
	R: 5′-TGGCATCAGCGCAGAGACT-3′
β-Actin	F: 5′-CGCTTCACGAATTTGCGTGTCAT-3′
	R: 5′-GCTTCGGCAGCACATATACTAAAAT-3′

### Western blot

After 4 and 6 weeks of myopia induction, 6 samples were randomly selected from each group. Phenylmethylsulfonyl fluoride-containing radioimmunoprecipitation assay buffer lysate was added at a mass to volume ratio of 10 mg:100 μl. The tissues were fully ground by electric homogenization at 4 °C for 120 s and centrifuged at 5,000 rpm for 5 min (NEST Biotechnology), after which the supernatants were collected. To identify the target protein, 10% sodium dodecyl sulfate‒polyacrylamide gel electrophoresis (Shandong Sparkjade Biotech. Co.) was used to separate the target proteins, and a polyvinylidene difluoride (PVDF) membrane was used for membrane transfer. The primary antibodies were used to analyze the target protein expression of the target proteins (Table 2). Primary antibodies were incubated with the membranes overnight at 4 °C, and then the PVDF membrane-loaded target proteins were incubated with secondary antibodies for 1 h at 4 °C. Finally, images were acquired using the FUSION-FX7 imaging system (Vilber Lourmat) and quantified using Fusion CAPT software (Vilber Lourmat).

**Table 2:** List of primary antibodies.

Primary antibody	Dilution	Lot number and manufacturer
GPC6, glypican 6	1:800	PAB40689, Bioswamp
ERBB4, Erb-B2 receptor tyrosine kinase 4	1:800	a6133, ABclonal
NLGN1, neuroligin 1	1:1,000	a16105, ABclonal
MAGI2, membrane-associated guanylate kinase inverted 2	1:10,000	25189-1-AP, Proteintech
PSD95, postsynaptic density protein 95	1:500	a6194, ABclonal
IL1RAPL1, interleukin-1 receptor accessory protein-like 1	1:1,000	bs-0445R, BIOSS
Bcl-2	1:2,000	12789-1-AP, Proteintech
Bax	1:5,000	50599-2-Ig, Proteintech
β-Actin	1:5,000	bs-10966R, Bioss

### TUNEL assay

Eyeballs were fixed in eye fixative (G1109, Servicebio) for 1 h at room temperature, and then the slices were treated with 0.5% Triton X-100 for 10 min. After being washed with PBS, proteinase K solution was subsequently added and the slices were subsequently incubated with the In Situ Cell Death Assay kit (Roche) according to the manufacturer’s instructions. Nuclei were costained for 5 min with 0.1 g/ml 4,6-diamidino-2-phenylindole (DAPI) (Beyotime). Finally, the images were captured under a fluorescence microscope (Zeiss, AX10) and cells positive for TUNEL were counted using the following formula: TUNEL/DAPI × 100%.

### Immunohistochemical staining

The eyeballs were fixed in eye fixative (G1109, Servicebio). The paraffin slices were placed in 3% hydrogen peroxide solution and incubated at room temperature in the dark for 25 min, after which the slides were placed in PBS (pH 7.4) and washed for 5 min three times on a decolorizing shaking table. The tissue was uniformly covered with 3% BSA in the chemical ring and sealed at room temperature for 30 min. The sealing solution was gently removed, PBS was added to the slices with an appropriate amount of primary antibody (Table 2) and the slices were placed flat in a wet box at 4 °C for overnight incubation. The slides were placed in PBS (pH 7.4) and washed by shaking on a decolorizing shaker three times for 5 min each. After the slices were slightly dried, the tissue was covered with the secondary antibody (HRP label) of the corresponding species of the primary antibody and incubated at room temperature for 50 min. Finally, the sections were observed under a white light microscope (E100, Nikon).

### Immunofluorescence staining

The same procedure was performed as described in ‘Immunohistochemical staining’ section. After the secondary antibody was added, the slides were placed in PBS (pH 7.4) and washed by shaking on a decolorizing shaker for 5 min three times. Next, a DAPI dye solution was added and the slides were incubated at room temperature in the dark for 10 min. The slides were then washed for 5 min three times in PBS (pH 7.4) on a decolorizing shaker. A quenching agent B solution was subsequently added for 5 min, after which the slides were rinsed with water for 10 min. Finally, the slides were sealed and observed under a fluorescence microscope (Nikon Eclipse, 55i).

### Golgi staining

The brain tissues were fixed in 4% paraformaldehyde and cut into 2–3-mm-thick slices. The slices were subsequently subjected to Golgi staining in a ventilated protected from light for 14 days. After staining, the tissue treatment mixture was changed for 1 h, followed by another change at 4 °C and the samples were kept in the dark for 3 days. Next, the brain tissue was securely attached to the tray of a vibrating microtome (Leica, VT1000S) using 502 super glue and immersed in the tissue treatment solution. The tissues were sliced into 60-μm-thick sections and each section was then carefully placed onto a slide with a brush, followed by washing with ultrapure water and treatment with Golgi development solution for 30 min. Next, the slices were sealed with glycerin gelatin and stored at room temperature in a section box in the dark. Finally, a digital tissue section scanner (3DHISTECH, Pannoramic MIDI) was used to scan and observe the slices.

### H&E staining and Nissl staining

For hematoxylin and eosin (H&E) staining, paraffin sections were dewaxed and stained with an eosin dye solution, followed by dehydration and sealing. First, the sections were treated with HD constant staining pretreatment solution for 1 min. The sections were then immersed in hematoxylin solution for 3–5 min and rinsed with tap water. Next, the sections were treated with hematoxylin differentiation solution and hematoxylin bluing solution, followed by another rinse with tap water. Finally, the sections were placed in 95% ethanol for 1 min and then stained with eosin dye for 15 s. Measurement of retinal layer thicknesses and ganglion cell layer thickness in the ventro-dorsal and naso-temporal axes from 1 to 5 mm from the optic rim was conducted at 4 and 6 weeks of the experiment.

For Nissl staining, the spinal specimens were fixed with formaldehyde, embedded in paraffin and then cut into 4-μm-thick sections. These sections were deparaffinized with xylene and rehydrated through a graded series of alcohols. Next, the sections were treated with Nissl staining solution (Servicebio) for 5 min and then mounted with neutral balsam. Finally, the sections were observed under a light microscope (Nikon, Eclipse E100) and representative images were captured using a camera (Nikon, Nikon DS-U3). The mean number of RGC neurons was calculated to assess neuronal loss.

### Statistical analysis

Statistical analyses were performed using GraphPad Prism (v.10.1.2) (for experimental data), R (v.4.3.2) and RStudio (v.3.5.3). Conservations between cell types were assessed by Pearson correlation analysis on the basis of the mean UMI of expressed genes in each cell cluster, and functional enrichment analysis of DEGs was performed using the Database for Annotation, Visualization, and Integrated Discovery (DAVID). For differential expression analysis in Seurat, unpaired and two-tailed Student’s *t*-tests were used to analyze data between the four groups. *P* < 0.05 was considered significantly different. Unless otherwise noted, each experiment was repeated three or more times with biologically independent samples.

## Results

### RGC apoptosis occurs in myopia

**Fig. 1:**
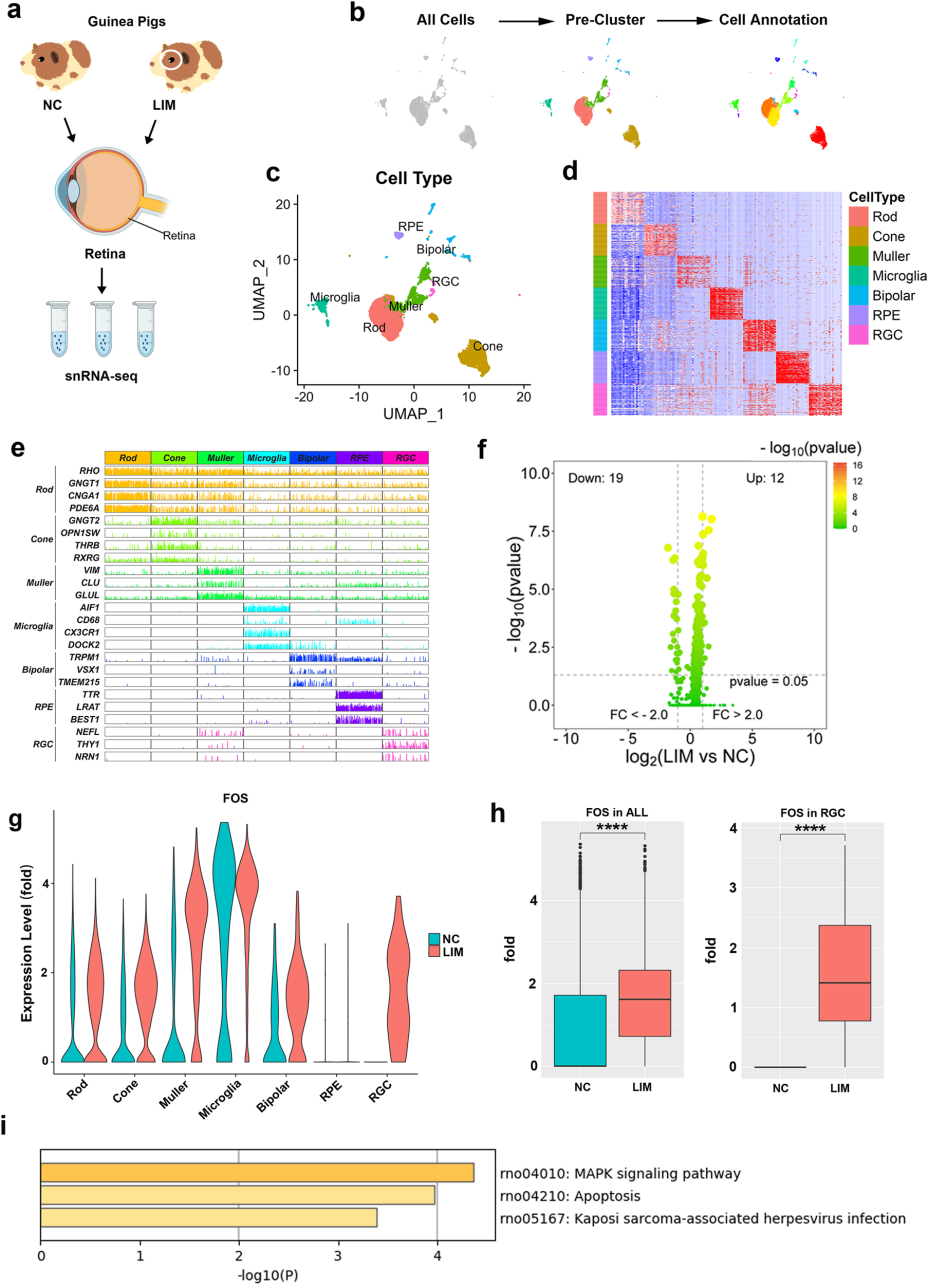
Retinal scRNA-seq in myopia. **a** ScRNA-seq of guinea pig retinal tissues in the NC and LIM groups (*n* = 3). **b** Cell clustering and annotation process. **c** UMAP visualization of the transcriptomic diversity of the retina. Cell types are shown in different colors (*n* = 3). **d** Normalized expression of each gene within each cell type. In both images, the rows correspond to cell types and columns to genes. **e** Expression of canonical markers of each cell type. **f** Volcano map of DEGs in RGCs of the LIM group versus the NC group. **g** Fos expression in cell types. **h** Fos expression levels in all retinal cells and RGCs. **i** Differential KEGG enrichment analysis of RGCs in the LIM group.

After 4 and 6 weeks of myopia induction, the myopic diopter and axial length increased significantly in the LIM group (all *P* < 0.001) (Supplementary Fig. 1). We performed scRNA -seq on the retinas of myopic guinea pigs and NCs (Fig. 1a). A total of 5,296 cells from myopic retinas and 3,528 cells from NC retinas were analyzed. On the basis of the currently available literature, the gene markers of different retinal cells in humans and mice were compiled and used to annotate guinea pig retinal cells (Fig. 1b). We identified seven cell types in the guinea pig retina, namely, photoreceptor cells (rods and cones), RGCs, bipolar cells, retinal pigment epithelium cells (RPEs) and glial cells (Müller glia and microglia) (Fig. 1c–e). On this basis, we focused on the analysis of the top 20 DEGs (HSPB1, ZFP36, ENSCPOG00000015206, GADD45B, ENSCPOG00000037287, EBF1, CRYAB, ENSCPOG00000032637, FOS, RPL12, DNAJB1, ACTB, ENSCPOG00000010438, Gapdh, ENSCPOG00000007441, ENSCPOG00000024660, BTG2, RELB, UBB and ENSCPOG00000027474) in RGC cells (Fig. 1f), and FOS expression increased significantly in rod, cone, Müller, microglia, bipolar and RGC cells (Fig. 1g, h). The top 20 DEGs were related to apoptosis signaling pathways (Fig. 1i).

**Fig. 2:**
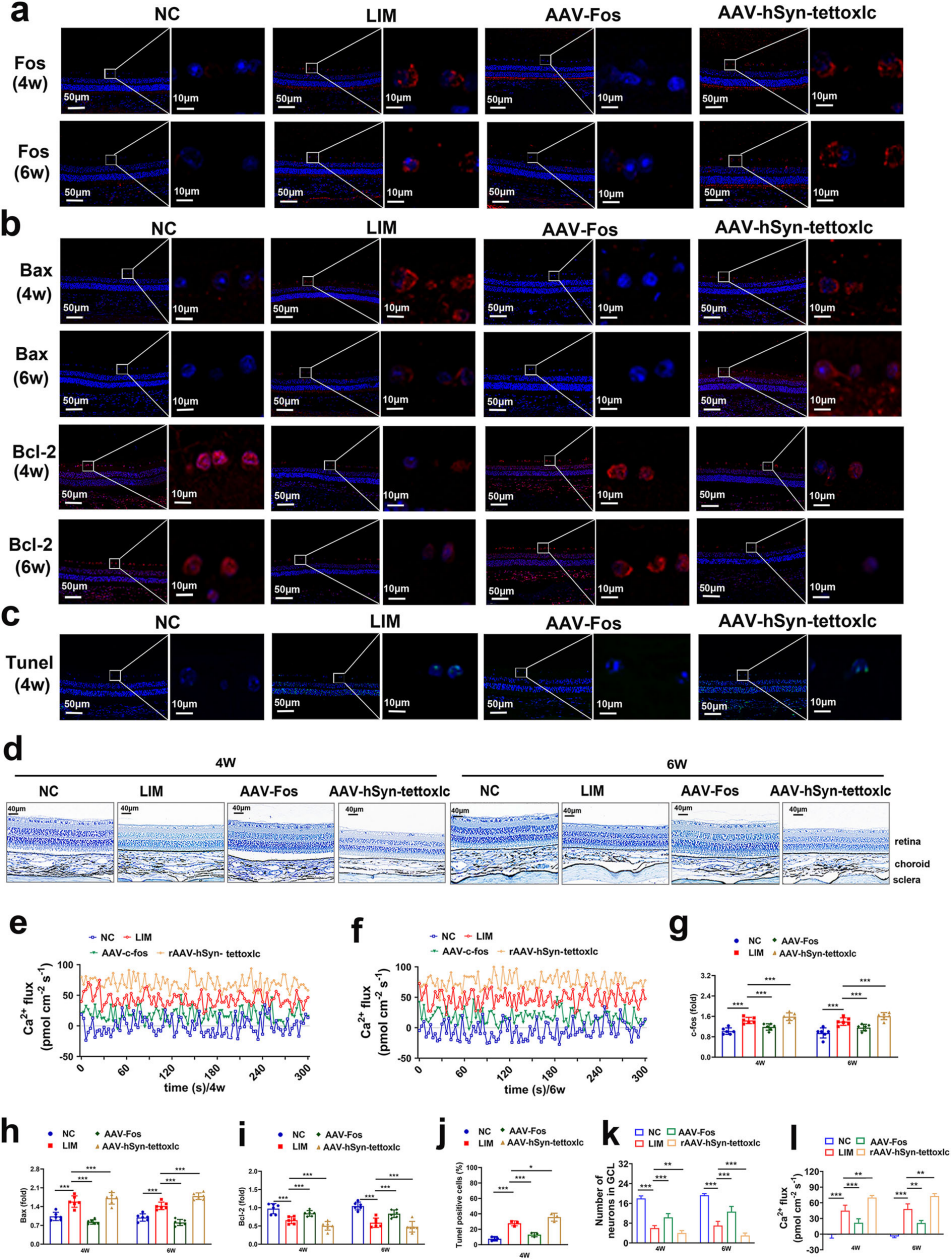
Elevated RGC apoptosis in experimental myopia. **a** FOS staining of the retina after myopic induction for 4 weeks (4w) and 6 weeks (6w). Green fluorescence represents apoptotic cells. **b** Bax and Bcl-2 staining of the retina after myopic induction for 4 and 6 weeks. Red fluorescence represents apoptotic cells. **c** TUNEL staining of the retina after myopic induction for 4 weeks. Green fluorescence represents apoptotic cells. **d** Retinal Nissl staining. Red arrows indicate RGCs and neuron particles are in dark blue (**n** = 3). **e**, **f** The 5 min data of Ca^2+^ in the retina recorded by NMT after myopic induction for 4 (**e**) and 6 (**f**) weeks (*n* = 4). **g** Bar graphs of FOS levels after myopic induction for 4 and 6 weeks (***P < 0.001) (*n* = 3). **h**, **i** Bar graphs of Bax (**h**) and Bcl-2 (**i**) levels after myopic induction for 4 and 6 weeks (*n* = 3). **j** Bar graphs of apoptosis percentage according to the TUNEL analysis after myopic induction for 4 weeks (*n* = 3). **k** Bar graphs of Nissl staining results of RGCs at 4 and 6 weeks after myopia induction (**P < 0.01) (*n* = 3). **l** Analysis of the retinal Ca^2+^ based on NMT for 4 weeks (***P < 0.001) (*n* = 4).

In addition, compared with the NC group, immunofluorescence analysis revealed that Fos expression was greater in the LIM group. Compared with the LIM group, the expression of Bax was decreased in the AAV-Fos group, whereas the expression of Fos was increased in the AAV-hSyn-tettoxlc group (*P* < 0.001) (Fig. 2a, g). Compared with the NC group, Bcl-2 expression decreased and Bax expression increased in the LIM group, and compared with the LIM group, Bcl-2 expression increased and Bax expression decreased in the AAV-Fos group, whereas Bcl-2 expression decreased and Bax expression increased in the AAV-hSyn-tettoxlc group (all *P* < 0.001) (Fig. 2b, h, i). Similarly, TUNEL detection revealed that the apoptotic ratio of RGCs in the LIM group was significantly greater than that in the NC group. However, compared with the LIM group, the apoptotic ratio of RGCs was lower in the AAV-Fos group and greater in the AAV-hSyn-tettoxlc group (Fig. 2c, j). Next, the experimental myopic RGCs were further analyzed for validation. Nissl staining revealed that the number of RGCs was lower in the LIM group than in the NC group, while compared with the LIM group, the number of RGCs in the AAV-Fos group was significantly greater, and the number of RGCs in the AAV-hSyn-tettoxlc group was significantly lower (Fig. 2d, k). NMT was used to measure Ca^2+^ flux with a selective Ca^2+^ microsensor on the inner surface of the retina after myopic induction (Fig. 2e, f). The results revealed that the Ca^2+^ fluxes measured at 4 and 6 weeks in the LIM group tended to outflow; compared with the LIM group, the Ca^2+^ flux outflows in the AAV-Fos group were significantly lower and the Ca^2+^ flux outflows in the AAV-hSyn-tettoxlc group were considerably greater (all *P* < 0.001) (Fig. 2l).

**Fig. 3:**
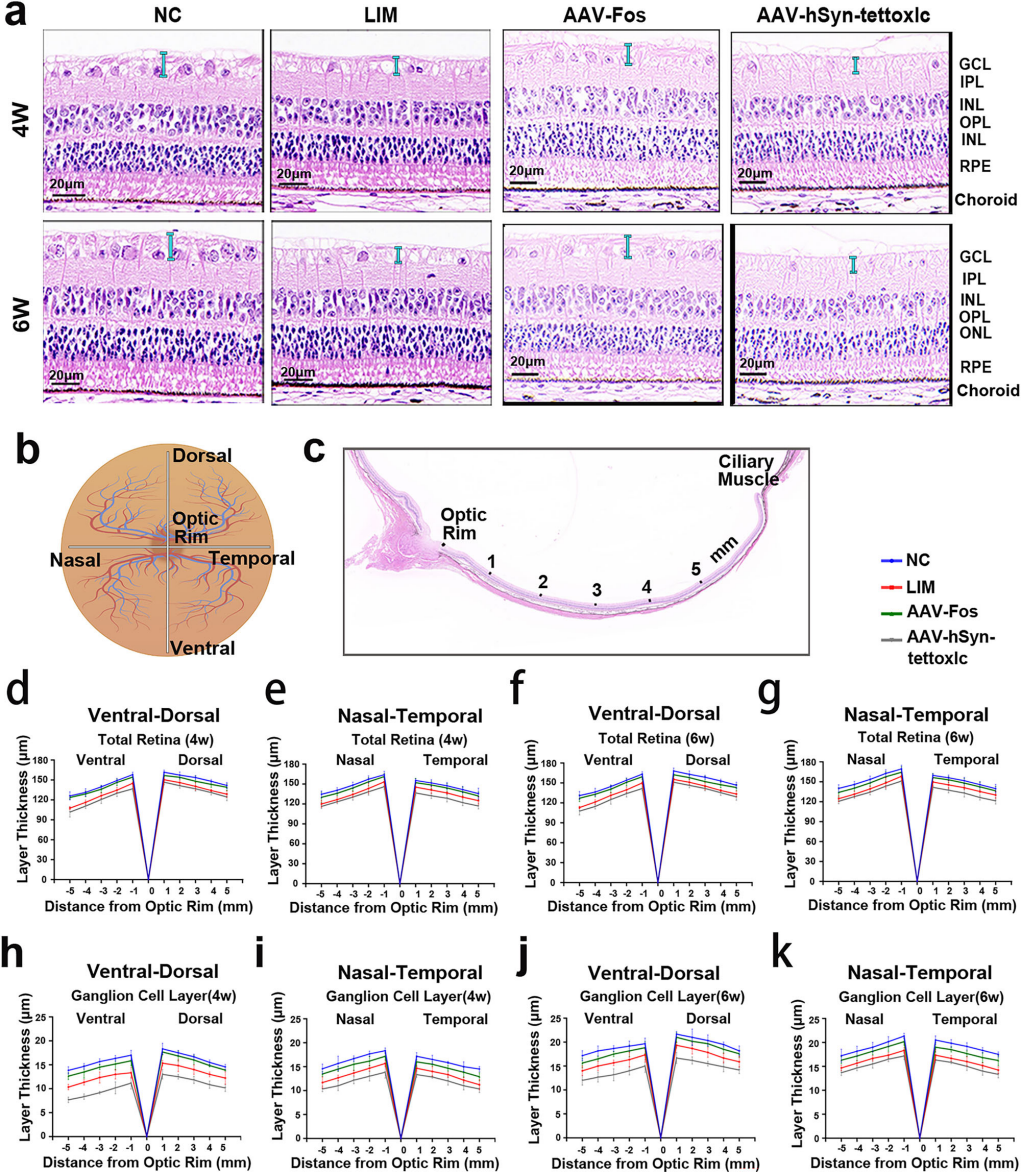
Spider graphs illustrating retinal thicknesses. **a** H&E staining of the retina. Sections marked in blue are the tissue structure of RGCs (1 mm from the optic rim). **b** A schematic fundus image illustrating the various planes along which the retinal thickness is measured. **c** A diagram of measurement locations. **d**–**k** Retinal layer thicknesses and ganglion cell layer thickness in the ventro-dorsal (**d**, **f**, **h** and **j**) and naso-temporal (**e**, **g**, **i** and **k**) measured from 1 to 5 mm from the optic rim at 4 weeks (**d**, **e**, **h** and **i**) and 6 weeks (**f**, **g**, **j** and **k**) of the experiment, showing the TR thickness (**d**–**g**) and the nerve fiber layer thickness (**h**–**k**).

H&E staining was performed to assess retinal thickness. Measurements were conducted along the vertical plane (Fig. 3a) and horizontal planes (Fig. 3b, c), passing through the optic nerve at five distinct distances (1 mm, 2 mm, 3 mm, 4 mm and 5 mm) from the nerve head. In all four eccentricity positions, both TR and RGC layer thickness decreased progressively with increasing distance from the optic nerve edge (Fig. 3d–k). At the same location, the LIM group exhibited diminished TR thickness and Ganglion cell layer (GCL) thickness when observed alongside the NC group. Following synaptic transmission inhibition, both TR thickness and GCL thickness were noted to be thinner than those seen in the LIM group. After FOS expression suppression, both TR thickness and GCL thickness were observed to be greater than those in the LIM group (Fig. 3d–k).

### RGC apoptosis leads to inactivation of the visual pathway in myopia

**Fig. 4:**
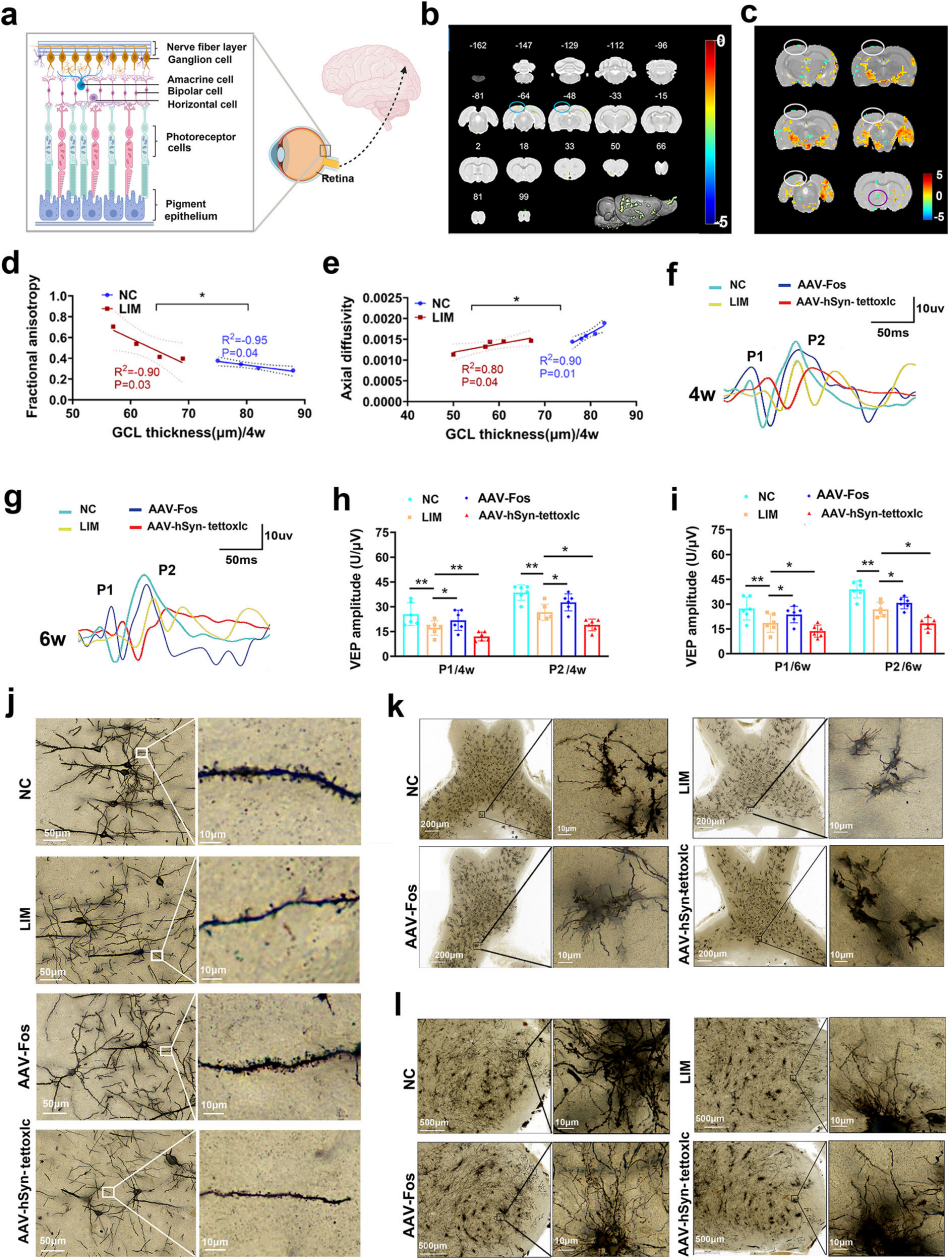
Visual circuit function analysis by FVEP, Golgi staining and fMRI. **a** Ganglion cells in the retinal tissue are the beginnings of the optic nerve, transmitting visual signals to the visual cortex. **b** FC maps of fMRI in the NC and LIM groups after myopic induction for 4 weeks. The blue circle represents the visual cortex of the target brain area. The deeper the green area, the lower the FC (*n* = 5). **c** Clusters with significant changes in ReHo values between NC and LIM groups after 4 weeks of myopic induction (false discovery rate (FDR) corrected, *P* < 0.05, cluster size >15 voxels). The white circle is the visual cortex of the target brain area. The red–yellow area indicates that the ReHo value of the LIM group is higher than that of the control group. The green area shows that the ReHo value of the LIM group is lower than that of the control group. **d**, **e** A linear correlation analysis between FA (**d**), AD (**e**) and RGC thickness in the DTI index detected by fMRI (*n* = 4-5). **f**, **g** The amplitude of the FVEP waveform after myopic induction for 4 weeks (**f**) and 6 weeks (**g**). **h**, **i** Bar graphs of FVEP P1 and P2 amplitude after myopic induction for 4 weeks (**h**) and 6 weeks (**i**) (***P < 0.001) (*n* = 4). **j** Golgi staining of the visual cortex. Local magnification shows the neuronal dendrites (*n* = 3). **k** Golgi staining of optic tract and optic chiasma. Local magnification shows the neuronal structure. **l**, Golgi staining of LGN. Local magnification shows the neuronal dendrites.

All of the brain’s information about the content of the visual world is based on the pulsed activity of the RGCs (Fig. 4a). We therefore examined visual pathways and visual cortex function. Compared with the NC group, DTI analysis revealed that the fractional anisotropy (FA) values of the optic nerve in the LIM group were significantly greater, whereas the axial diffusivity (AD) values of the optic tract and optic chiasma were significantly lower, and the mean diffusivity (MD), AD and radial diffusivity (RD) values of the posterior commissure were also significantly lower (all *P* < 0.05) (Table 3). Reduced FC was found between the visual cortex and the left (L)-hypothalamus, L-endo-olfactory cortex, L-periolfactory cortex and L-primary visual cortex (Table 4 and Fig. 4b). Compared with the NC group, the ReHo values of the optic tract and optic chiasm L, primary visual cortex binocular area L, primary visual cortex monocular area R, medio lateral secondary visual cortex L and primary auditory cortex L decreased in the LIM group (Table 5 and Fig. 4c), and the ReHo values of the superficial gray layer of the superior colliculus L, external cortex of the inferior colliculus R and dentate gyrus R increased.

**Table 3:** Mean diffusion parameters values of the NC and LIM groups.

	Brain clusters	NC	LIM
FA	Optic nerve	0.35 ± 0.02	0.48 ± 0.03*
MD	Postcommissure	0.0023 ± 0.0004	0.0014 ± 0.0005*
AD	Postcommissure	0.0029 ± 0.0007	0.0013 ± 0.0002***
	Optic tract and optic chiasma	0.0017 ± 0.0003	0.0013 ± 0.0002*
RD	Postcommissure	0.0022 ± 0.0002	0.0008 ± 0.0003***

*P < 0.05 and ***P < 0.001 compared with the NC group. FA, MD, AD and RD with s.d. based on both median and ulnar nerves in the NC and the LIM groups (*n* = 4–5).

($$\bar{{\rm{X}}}$$ ± s).

**Table 4:** Information of FC in multiple brain regions.

Condition	Brain clusters	Number of voxels	Peak intensity	Peak MNI coordinate
LIM < NC	Hypothalamus_L	51	5.11	−12 −31 −32
	Entorhinal cortex_L	19	5.76	−42 −16 −26
	Perolfactory cortex_L	16	4.30	−51 −97 31
	Primary visual cortex_L	10	4.29	−48 −49 55
	Primary ocellar area of visual cortex_L	53	6.6	−27 −67 58

For each voxel, comparison thresholds were *P < 0.05, FDR corrected and cluster size >10 voxels (*n* = 5.). *MNI* Montreal Neurological Institute, *L* Left.

**Table 5:** Activation information of ReHo in the multiple brain regions.

Condition	Brain clusters	Number of voxels	Peak intensity	Peak MNI coordinate
LIM ＞ NC	Superficial_gray_layer_of_the_superior_colliculus_L	198	4.4886	−13 −71 42
	External_cortex_of_the_inferior_colliculus_R	87	3.1057	14 −89 42
	Dentate_gyrus_R	168	6.2079	5 −26 33
LIM < NC	Optic_tract_and_optic_chiasm_L	16	−4.9242	5 34 −21
	Primary_visual_cortex_binocular_area_L	17	−4.0284	−49 −68 63
	Primary_visual_cortex_monocular_area_R	18	−3.9114	32 −59 60
	Medio_lateral_secondary_visual_cortex_L	23	−3.6491	−31 −44 69
	Primary_auditory_cortex_L	114	−4.184	−64 −38 21

For each voxel, comparison thresholds were *P < 0.05, FDR corrected and cluster size >15 voxels (*n* = 4–5). *LIM* Lens-induced myopia, *NC* Normal Control.

Furthermore, FA was negatively correlated with RGC thickness and AD was positively correlated with RGC thickness according to correlation analysis (all *P* < 0.05) (Fig. 4d, e).

Using the OPTOPROBE ophthalmic imaging system, the FVEP assay was performed at 4 and 6 weeks (Fig. 4f, g). The results revealed that P1 (NC versus LIM at 4 weeks: 25.77 ± 8.30 μV versus 18.28 ± 6.27 μV; 6 weeks: 26.72 ± 9.67 μV versus 17.67 ± 9.84 μV) and P2 (NC versus LIM at 4 weeks: 38.58 ± 8.27 μV versus 26.57 ± 6.80 μV; 6 weeks: 40.44 ± 9.56 μV versus 18 ± 8.22 μV) waveforms in the LIM group changed significantly, and the amplitude decreased significantly compared with that in the NC group. Compared with the LIM group, the P1 (NC versus LIM at 4 weeks: 18.28 ± 6.27 μV versus 38.43 ± 5.64 μV; 6 weeks: 17.67 ± 9.84 μV versus 23.05 ± 6.74 μV) and P2 (NC versus LIM at 4 weeks: 26.57 ± 6.80 μV versus 30.77 ± 7.43 μV; 6 weeks: 18 ± 8.22 μV versus 30 ± 9.31 μV) waveforms and the amplitudes were increased in the AAV-Fos group, whereas the P1 (NC versus LIM at 4 weeks: 18.28 ± 6.27 μV versus 12.05 ± 3.58 μV; 6 weeks: 17.67 ± 9.84 μV versus 13.05 ± 4.90 μV) and P2 (NC versus LIM at 4 weeks: 26.57 ± 6.80 μV versus 20.01 ± 5.22 μV; 6 weeks: 18 ± 8.22 μV versus 14 ± 5.05 μV) waveforms and the amplitudes were reduced in the AAV-hSyn-tettoxlc group (all *P* < 0.001 or *P* < 0.01) (Fig. 4h, i).

After 4 weeks of myopia induction, Golgi staining revealed that neuronal axonal and dendritic morphology in the LIM group was impaired and that the number of dendritic spines in the visual cortex was reduced compared with that in the NC group. Compared with the LIM group, the AAV-Fos group presented an intact neuron structure and an increased number of dendritic spines, whereas the AAV-hSyn-tettoxlc group presented a severely damaged structure and a decreased number of dendritic spines (Fig. 4j).

Golgi staining revealed that the structures of the neurons in the optic chiasma and LGN were damaged in the LIM group; compared with those in the LIM group, the structures of the neurons in the optic chiasma and LGN were relatively complete in the AAV-Fos group, and the structure was more severely damaged in the AAV-hSyn-tettoxlc group (Fig. 4k). Golgi staining revealed that the dendrite density of neurons in the LGN was decreased in the LIM group; compared with the LIM group, the dendritic density was increased in the AAV-Fos group and the dendritic density was decreased in the AAV-hSyn-tettoxlc group (Fig. 4l).

### The number of neurons and the expression of synaptic-related factors are decreased in the visual cortex in myopia

**Fig. 5:**
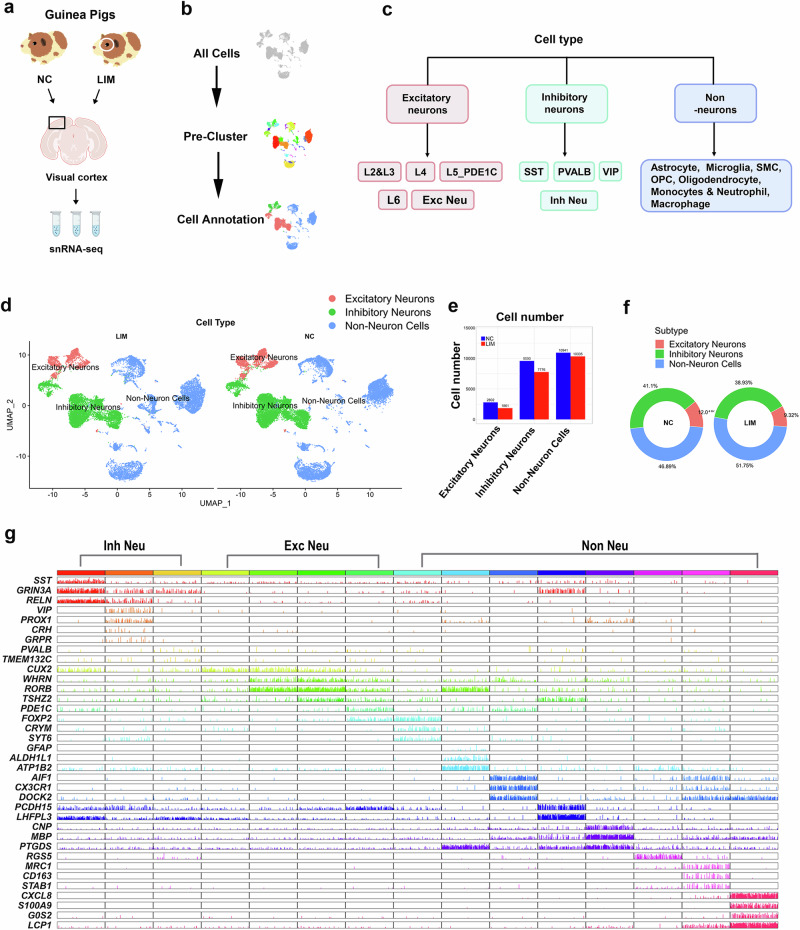
Visual cortex scRNA-seq in experimental myopia. **a** ScRNA-seq of visual cortex tissues in NC and LIM groups (*n* = 3). **b** Cell clustering and the annotation process. **c** Cellular taxonomy of the visual cortex. **d** UMAP visualization of visual cortex transcriptomic diversity. Cell types are shown in different colors for the three main cell classes. **e** Changes in the cell number in the three cell classes in the LIM group compared with the NC group. **f** Changes in the proportion of the three cell classes in the LIM group versus the NC group. **g** Differential gene expression confirmed the identification of cell marker genes for the three assigned cell types. The color bar contains the name and number of the core cell that represents the type (exc, excitatory; inh, inhibitory).

We performed snRNA-seq and scRNA-seq of the visual cortex of the experimental myopic guinea pigs. The cells from the LIM animals were well distinguished from those from the NC animals (Fig. 5a). In addition to unsupervised classification (uniform manifold approximation and projection (UMAP)), a previously established set of cell type-specific genes (included in the BRETIGEA software package) was used to identify cell types^19,20^. The nuclei were divided into clusters corresponding to the three main cell types established by McKenzie^19^ and Kelley^20^ in the brain, including glutamatergic neurons (excitatory neurons), GABAergic neurons (inhibitory neurons) and non-neurons, and these three cell types (Fig. 5b–d) were further classified into a total of 17 cell types (Supplementary Fig. 2).

We analyzed three classes (glutamatergic neurons, GABAergic neurons and non-neurons) and 17 cell types (Supplementary Fig. 2) in myopic samples compared with NCs. Compared with the NC group, the number (Fig. 5e) and proportion (Fig. 5f) of excitatory and inhibitory neurons in the LIM group were lower. In addition, we detected reductions in the number and proportion of Smooth muscle cell (SMCs) and macrophages across 17 cell types (Supplementary Fig. 2). To confirm the validity of the identified genes, we examined the genes that were differentially expressed between the cell types. We also reproduced the marker genes reported by Tasic et al.^21^ (Fig. 5g).

**Fig. 6:**
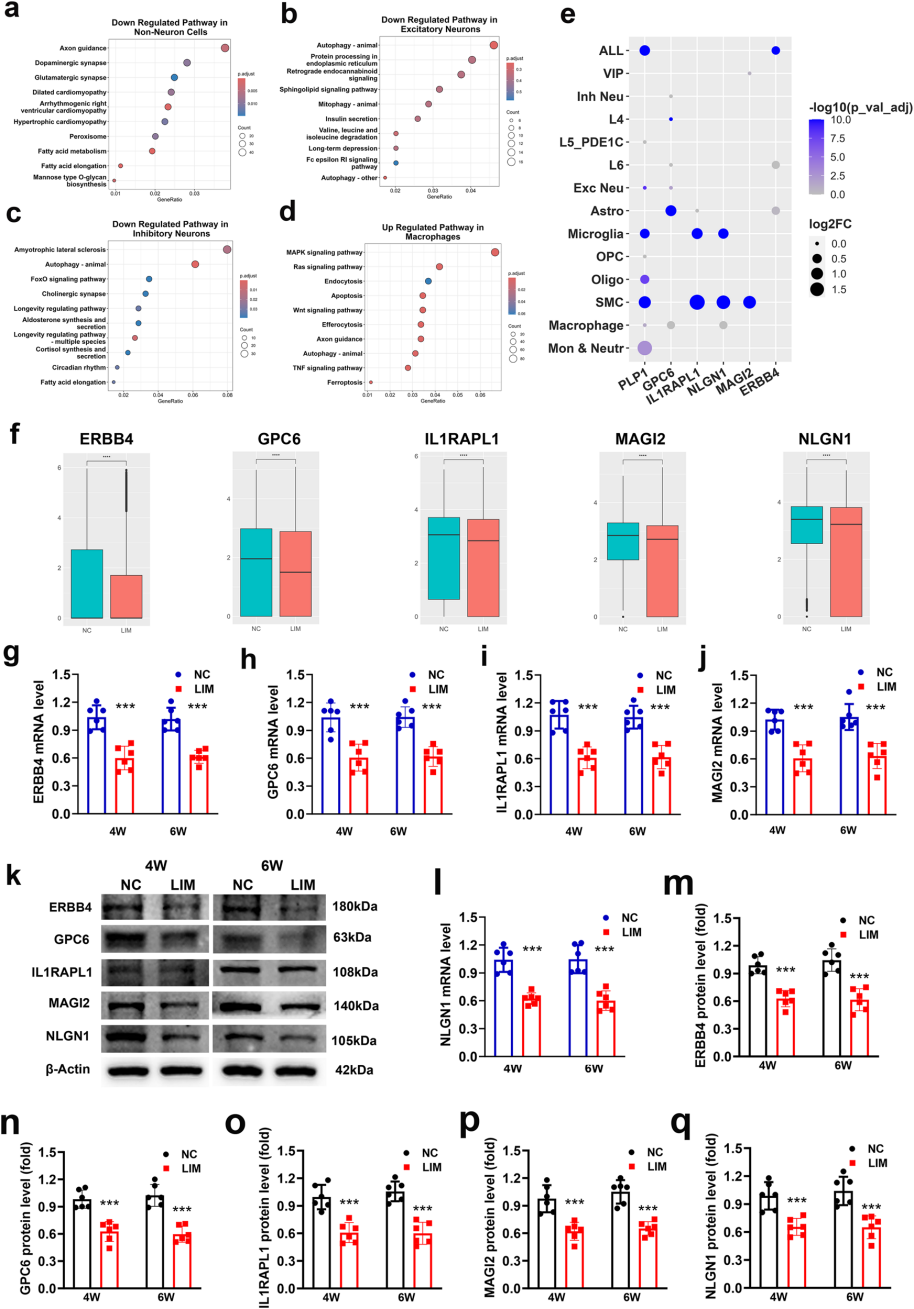
Visual cortex scRNA-seq and snRNA-seq in experimental myopia. **a**–**d** KEGG enrichment analysis of downregulated genes of non-neurons (**a**) excitatory neurons (**b**) and inhibitory neurons (**c**) and upregulated genes of macrophages (**d**) in the visual cortex of the LIM group. **e** Expression levels of synaptic-related genes (ERBB4, GPC6, IL1RAPL1, MAGI2 and NLGN1) in the visual cortex of the LIM group and NC group in different cells. **f** Expression levels of synaptic-related genes (ERBB4, GPC6, IL1RAPL1, MAGI2 and NLGN1) in the visual cortex of the LIM group and NC group in different cells. **g**–**j** Bar graphs of ERBB4 (**g**) GPC6 (**h**) IL1RAPL1 (**i**) and MAGI2 (**j**) expression detected by RT–qPCR in NC and LIM (***P < 0.001) (*n* = 6). **k** ERBB4, GPC6, IL1RAPL1, MAGI2 and NLGN1 expression detected by western blot in NC and LIM. Samples were derived from the same experiment and blots were processed in parallel (***P < 0.001) (*n* = 6). **l** A bar graph of NLGN1 expression detected by RT–qPCR in NC and LIM (***P < 0.001) (*n* = 6). **m**–**q** Bar graphs of western blot analysis for ERBB4 (**m**) GPC6 (**n**) IL1RAPL1 (**o**) MAGI2 (**p**) and NLGN1 (**q**) in NC and LIM (***P < 0.001) (*n* = 6). Log2FC refers to the fold change, OP Coligodendrocyte precursor cell, VIP Vasoactive Intestinal Peptide.

One study has shown that synapse-related gene expression decreases in the visual cortex in monocular deprivation models^22^. Differences in the gene expression of non-neurons (Fig. 6a), inhibitory neurons (Fig. 6b) and excitatory neurons (Fig. 6c) in the LIM and NC groups revealed that the synaptic vesicle circulation pathway was enriched in the top ten downregulated signaling pathways (Fig. 6d). In addition, the dysfunction of neurons stimulated the immune function of macrophages, resulting in an increased number of macrophages and enhanced endocytosis and efferocytosis, facilitating the phagocytosis of dysfunctional cells^23,24^. The expression of ERBB4, GPC6, IL1RAPL1, MAGI2 and NLGN1 was significantly decreased in all cell types (Fig. 6e, f). We validated the synapse-associated factors of the DEGs in the scRNA-seq and snRNA-seq analysis. The results revealed that the expression of ERBB4, MAGI2, GPC6, IL1RAPL1 and NLGN1 was lower in the visual cortex of the LIM groups than in that of the NC group (all *P* < 0.001) (Fig. 6g–q).

### Reduced excitability and abnormal synaptic remodeling of cortical neurons in myopia

**Fig. 7:**
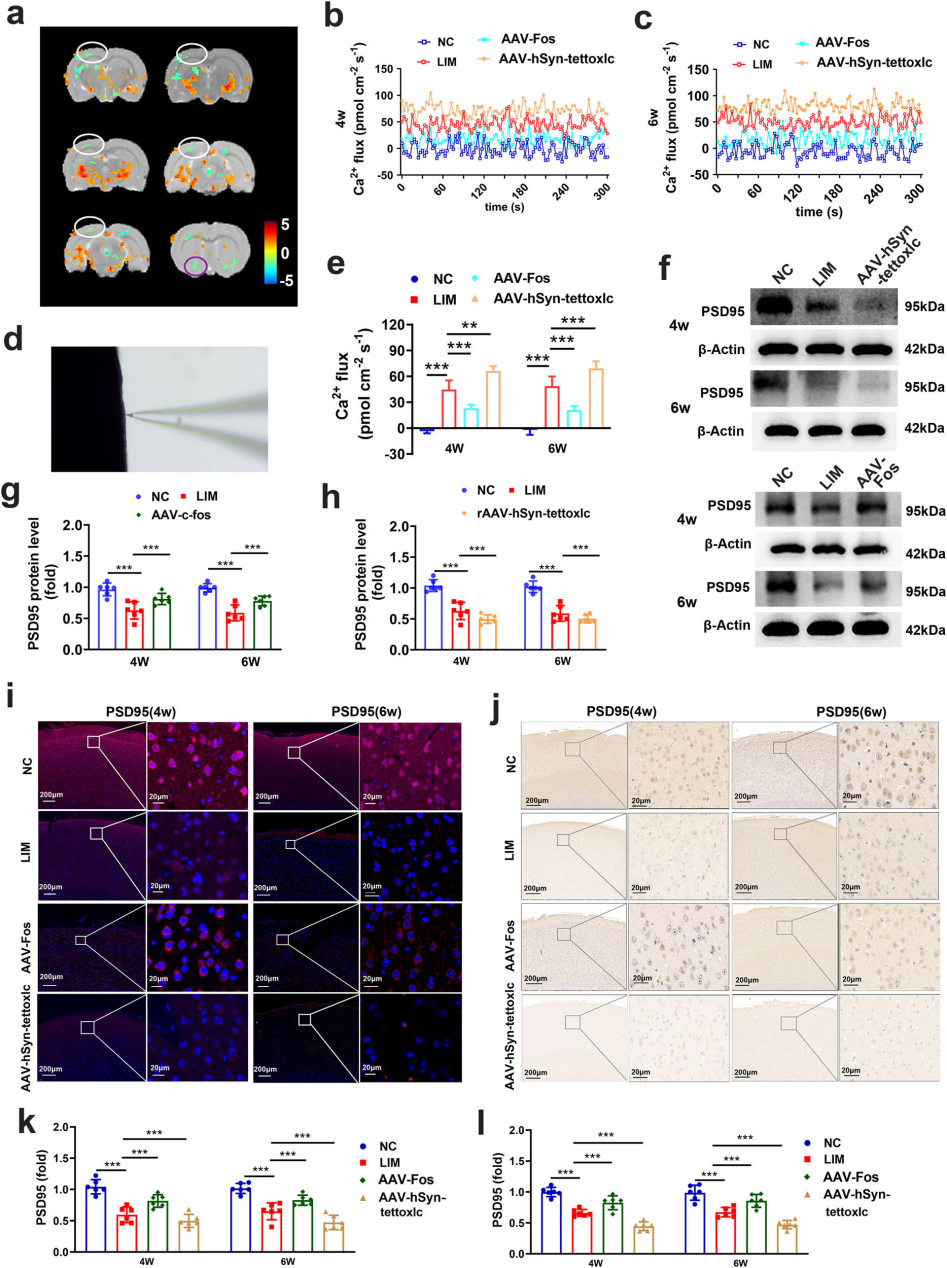
Neuronal excitability detection. **a** Clusters with significant changes in ALFF values between NC and LIM groups after 4 weeks of myopic induction (FDR corrected, *P* < 0.05, cluster size >15 voxels). The target brain regions are indicated by blue circles, the visual cortex by white circles and the optic tracts and chiasma are marked as purple circles. The red–yellow area indicates that the ALFF value of the LIM group is higher than that of the NC group. The green area demonstrates that the ALFF value of the LIM group is lower than that of the NC group. **b**, **c** The 5 min data analysis of [Ca^2+^] of the visual cortex recorded by NMT after 4 and 6 weeks of myopic induction (*n* = 4). **d** The NMT chart. **e** Analysis of retinal [Ca ^2+^] based on NMT (***P < 0.001) (*n* = 4). **f** PSD95 expression detected by western blot. The samples were derived from the same experiment and the blots were processed in parallel. **g**, **h** Bar graphs of western blot analysis for PSD95 at 4 weeks and 6 weeks. Samples were derived from the same experiment and blots were processed in parallel. **i**, PSD95 immunofluorescence staining. **j** PSD95 immunohistochemical staining. **k** Bar graphs of immunofluorescence staining for PSD95 (***P < 0.001) (*n* = 3). **l** Bar graphs of immunohistochemistry staining for PSD95 (***P < 0.001) (*n* = 3).

The ALFF values of primary visual cortex monocular area L, basal forebrain region L, primary somatosensory cortex barrel field L, primary visual cortex L, primary motor cortex L, primary visual cortex R, primary visual cortex binocular area R, and optic tract and optic chiasm L were significantly lower in the LIM group, while the ALFF value of external cortex of the inferior colliculus L was significantly greater in the LIM group than in the NC group. The details of the results are presented in Table 6 and Fig. 7a. NMT was applied to measure the Ca^2+^ flux of the surface of the inner retina (Fig. 7b–d). Ca^2+^ fluxes measured after 4 and 6 weeks of myopic induction in the NC group tended to reach a steady flux state near the baseline (4 weeks: −0.51 ± 12.54 pmol/cm^2^/s; 6 weeks: −5.55 ± 16.04 pmol/cm^2^/s), whereas Ca^2+^ fluxes measured in the LIM group tended to outflow (4 weeks: 42.74 ± 17.67 pmol/cm^2^/s; 6 weeks: 39.23 ± 15.84 pmol/cm^2^/s). Compared with those in the LIM group, the outflows of Ca^2+^ fluxes in the AAV-Fos group decreased (4 weeks: 20.33 ± 11.24 pmol/cm^2^/s; 6 weeks: 16.47 ± 8.67 pmol/cm^2^/s) and increased in the AAV-hSyn-tettoxlc group (4 weeks: 60.31 ± 15.31 pmol/cm^2^/s; 6 weeks: 56.89 ± 12.64 pmol/cm^2^/s) (*P* < 0.001; Fig. 7e). In addition, the levels of the excitatory synaptic marker PSD95 were significantly decreased in the visual cortex of myopic guinea pigs after 4 and 6 weeks of myopic induction. Compared with the LIM group, the expression of PSD95 was significantly increased in the AAV-Fos group and decreased in the AAV-hSyn-tettoxlc group (Fig. 7f–l). The results of fMRI, NMT and scRNA-seq in the visual cortex revealed that the excitability of neurons in the visual cortex was reduced in myopia.

**Table 6:** Activation information of ALFF in multiple brain regions of different treatment groups.

Condition	Brain clusters	Number of voxels	Peak intensity	Peak MNI coordinate
LIM ＞ NC	External_cortex_of_the_inferior_colliculus_L	68	4.1973	−8 −89.05 45.2
LIM < NC	Primary_visual_cortex_monocular_area_L	22	−4.0658	−38 −68.05 60.2
	Basal_forebrain_region_L	19	−2.9104	17.8 −29 6.95
	Primary_somatosensory_cortex_barrel_field_L	363	−5.4205	0.20 −53 −29.05
	Primary_visual_cortex_L	363	−5.4205	0.20 −53 −29.05
	Primary_motor_cortex_L	160	−2.8347	42.2 −20 39.95
	Primary_visual_cortex_R	24	−4.6341	49 −53.05 63.2
	Primary_visual_cortex_binocular_area_R	120	−3.0927	54.2 28 −65.05
	Optic_tract_and_optic_chiasm_L	16	−2.9104	−29 6.95 −14.8

For each voxel, comparison thresholds were *P < 0.05, FDR corrected and cluster size >15 voxels (*n* = 5), *ALFF* amplitude of low-frequency fluctuation, *LIM* Lens-induced myopia, *NC* Normal Control.

**Fig. 8:**
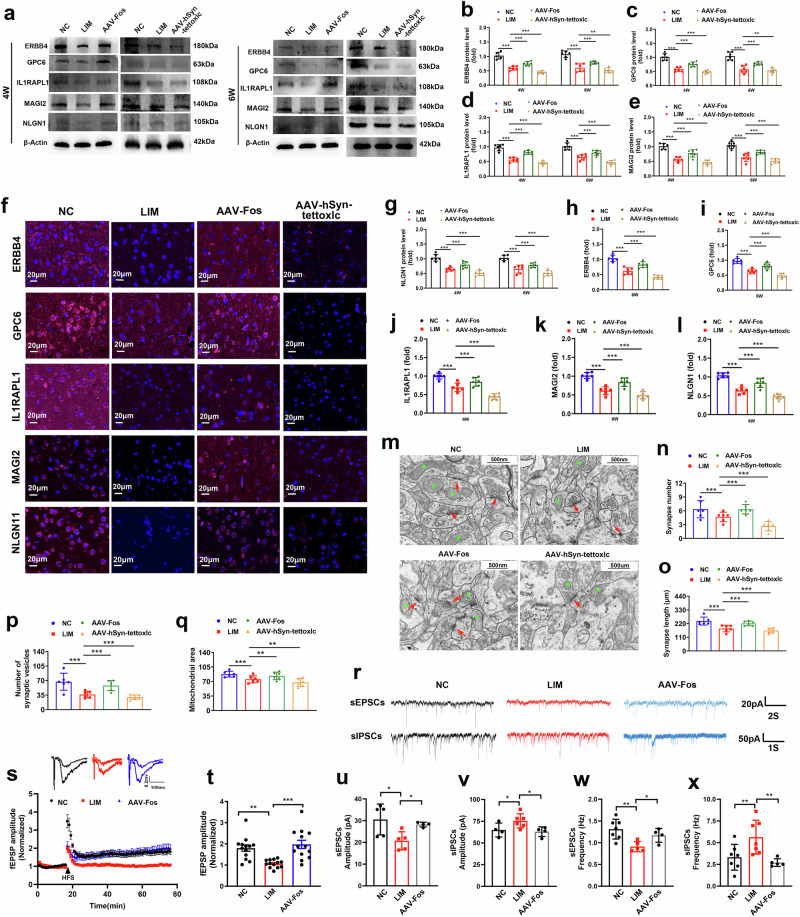
Synaptic morphology and function detection. **a** ERBB4, GPC6, IL1RAPL1, MAGI2 and NLGN1 expression detected by western blot after myopic induction for 4 and 6 weeks in the NC, LIM, AAV-Fos and AAV-hSyn-tettoxlc groups. The samples were derived from the same experiment and the blots were processed in parallel. **b**–**e**, Bar graphs of western blot analysis for ERBB4 (**b**), GPC6 (**c**), IL1RAPL1 (**d**) and MAGI2 (**e**) (***P < 0.001) (*n* = 6). **f** ERBB4, GPC6, IL1RAPL1, MAGI2 and NLGN1 expression were detected by immunofluorescence staining. **g** A bar graph of western blot analysis for NLGN1 (***P < 0.001) (*n* = 6). **h**–**l** Bar graphs of immunofluorescence staining for ERBB4 (**h**) GPC6 (**i**) IL1RAPL1 (**j**), MAGI2 (**k**) and NLGN1 (**l**) (***P < 0.001) (*n* = 3). **m** The synapses and mitochondria of the NC, LIM, AAV-Fos and AAV-hSyn-tettoxlc groups were detected by TEM. The red arrows indicate synapses and the green asterisk indicates mitochondria. **n**, A bar graph showing the number of synaptic vesicles detected by TEM (*P < 0.05) (*n* = 3). **o** A bar graph showing the synaptic length detected by TEM (*P < 0.05) (*n* = 3). **p** A bar graph showing the number of synaptic vesicles detected by TEM (*P < 0.05) (*n* = 3). **q** A bar graph showing the mitochondrial area detected by TEM (*P < 0.05) (*n* = 3). **r** The sEPSCs and sIPSCs amplitude and frequency recordings of the visual cortex were examined by the patch-clamp technique. **s** fEPSP amplitude recordings of the visual cortex examined by electrophysiological detection. **t** A bar graph of the of LTP of fEPSP amplitude. (***P < 0.001 or **P < 0.01) (*n* = 13 neurons/5 guinea pigs). **u**–**x** Bar graphs of sEPSCs (**u** and **w**) and sIPSCs (**v** and **x**) amplitude (**u** and **v**) and frequency (**w** and **x**) detected by the patch-clamp technique (*P < 0.05) (*n* = 4–7).

Synapses are essential hubs for the transmission of information between neurons. The results of western blot (Fig. 8a–e,g) and immunofluorescence (Fig. 8f, h–l), RT‒qPCR (Supplementary Fig. 3a–f) and immunohistochemical (Supplementary Fig. 3g–p) analyses revealed that the expression of ERBB4, MAGI2, GPC6, IL1RAPL1 and NLGN1 was lower in the visual cortex in the LIM groups than in the NC group. Compared with the LIM group, the expression of ERBB4, MAGI2, GPC6, IL1RAPL1 and NLGN1 was higher in the AAV-Fos group and lower in the AAV-hSyn-tettoxlc group (all *P* < 0.001). In this study, we focused on ultrastructural changes in synapses in the visual cortex region. We found that the synaptic density, length, synaptic gap, mitochondrial volume and number of presynaptic vesicles were significantly decreased in the LIM group. Compared with the LIM group, the synaptic density, length, synaptic gap, mitochondrial volume and number of presynaptic vesicles were significantly increased in the AAV-Fos group and decreased in the AAV-hSyn-tettoxlc group (Fig. 8m–q) (*P* < 0.001).

Whole-cell patch-clamp recordings targeted to the visual cortex were used to detect sEPSCs and sIPSCs (Fig. 8r). We found that sEPSC amplitude and frequency were significantly lower and that sIPSC amplitude and frequency were significantly greater in the LIM group than in the NC group. Compared with the LIM group, the sEPSC amplitude and frequency in the AAV-Fos group were significantly greater, whereas the sIPSC amplitude and frequency were significantly lower (Fig. 8u–x) (*P* < 0.05 or *P* < 0.01).

In addition, compared with the NC group, the amplitude of LTP in the model group was significantly lower and that in the AAV-Fos group was significantly greater than that in the LIM group (Fig. 8s, t). These results indicate that the synaptic transmission function of the myopic cortex is impaired and that synaptic transmission activity is weakened, suggesting that synaptic plasticity is abnormal and that Fos knockdown can inhibit the above phenomenon.

These findings indicate that the frequency of excitatory presynaptic and postsynaptic release in the visual cortex of the myopic group decreased, whereas the frequency of inhibitory presynaptic and postsynaptic vesicle release increased, indicating a decrease in excitatory projection synaptic transmission of visual cortex neurons and an increase in inhibitory projection synaptic transmission. Conversely, inhibiting RGC apoptosis inhibited TSD and abnormal synaptic remodeling, thereby inhibiting the development of myopia (Supplementary Fig. 1).

## Discussion

### Anterograde TSD occurs in the visual pathway in myopia

Anterograde TSD refers to the degeneration and necrosis of upstream neurons, and the ‘deprived’ isolation of downstream neurons is due to the lack of synaptic transmission signals, ultimately leading to degeneration and atrophy of downstream neurons^8,9^.

A previous study has shown that thinning of the peripapillary retinal nerve fiber layer causes a reduction in the volume of the visual cortex, suggesting that TSD contributes to chronic axon damage in multiple sclerosis^25^. After the onset of optic neuritis, damage to the radiating tissue of the optic nerve worsens. fMRI evidence shows a decrease in FA of the optic radiation and RD^26^. Therefore, the apoptosis, atrophy and inflammation of RGCs can lead to structural and functional degeneration of the visual cortex. In the present study, we found that the apoptosis of RGCs in myopia leads to structural and functional impairments in the downstream visual pathway and that knocking down FOS can inhibit the apoptosis of the retina and alleviate damage to the downstream visual pathway.

In addition, we found that the inhibition of synaptic transmission can exacerbate the development of myopia, supporting the hypothesis of anterograde TSD in myopia.

Vision occurs when visual information forms visual nerve impulses in the retina, which are transmitted by photoreceptor cells, bipolar cells and ganglion cells that transmit information to the visual center along the optic pathway. In this study, we found that the apoptosis of photoreceptor cells, bipolar cells and ganglion cells occurred in myopic guinea pigs. We found that Müller cells were also apoptotic in myopia. Studies have shown that gliosis and inflammation of Müller cells can lead to the apoptosis of RGCs^27,28^. Restoring the normal function of Müller cells can inhibit RGC damage in glaucoma^29^. Subretinal injection of AAV-GFAP-CasRx-Ptbp1 can specifically transform retinal Müller cells into RGCs, and the transformed ganglion cells can establish functional connections with the corresponding brain regions^30^, indicating that Müller cells are closely related to RGCs.

One study reported that Outer nuclear lay (ONL) and Inner nuclear lay (INL) as well as nerve fiber layer thickness were significantly lower in patients with myopia than in patients with normal eyes^31^. Another study has shown that, in myopia, the density of photoreceptor cells decreases with the length of the axis of the eye^32^, accompanied by a reduction in the thickness of the ONL^33^. Additionally, it has also been shown that a reduction in the thickness of the photoreceptor layer not only indicates the loss of photoreceptor cells but may also serve as an early indicator of retinal degenerative diseases, which can ultimately affect overall visual function and may trigger degenerative changes in retinal layers, such as RGCs, leading to a chain reaction^34^. Therefore, a decrease in the thickness of the photoreceptor layer could be one of the early signs of myopia and may provide insights into subsequent RGC damage in myopia. Fos activation may be closely related to the death or apoptosis of photoreceptor cells^35^. In this study, we found that both photoreceptor cells and RGC cells underwent apoptosis at 4 and 6 weeks of myopia, while knockdown of FOS significantly reduced apoptosis in the retina and inhibited myopia progression to some extent. However, these current data cannot definitively confirm whether photoreceptors or RGCs are the primary drivers of myopia development. We plan to employ more refined technical approaches in future studies to further validate the specific roles of photoreceptors and RGCs in myopia and elucidate their interrelationships in this condition.

RGCs are the only projection neurons in the neural retina. RGCs are the starting point of the optic nerve, and visual information is transmitted from the eye to the advanced processing center of the brain via the optic nerve, a bundle of axons that emerge from the output neurons of the retina and terminate in the LGN^36,37^. Therefore, RGCs were the main focus of this study. Increased Fos expression can promote retinal apoptosis^38–40^. Our study revealed that RGC apoptosis in experimental myopia decreased Bcl-2, increased Bax and induced retinal Ca^2+^ outflow. Previous studies have confirmed the apoptosis of RGCs^6^ and the thinning of the RGC layer in myopia^41^. Ca^2+^ is a highly versatile intracellular signal responsible for regulating a range of cellular processes, and the loss of Ca^2+^ homeostasis, usually in the form of cytoplasmic increases, leads to cell damage. Thus, a disturbance of calcium homeostasis is associated with RGC death^42,43^, and RGC death is related to a decrease in Bcl-2 and an increase in Bax in myopia^44,45^. Our results are consistent with the above findings, suggesting that RGCs in myopia are apoptotic and that knocking down Fos can inhibit Fos expression and retinal apoptosis.

In the present study, we observed dysfunction of the visual cortex in myopia via fMRI analysis. Typically, approximately 50% of human optic nerve fibers cross the human chiasma^46^. However, significant interspecies differences exist. For example, Polyak reported that guinea pigs have as little as 1% of optic fibers that do not cross^47^. The proportion of optic nerve fibers in mice at optic intersections is estimated to be between 90% and 95% (ref. ^48^). Therefore, in this study, we performed negative lens-induced myopia on the right eye of guinea pigs, with a focus on detecting and analyzing changes in the left cerebral visual cortex^49^. A previous study has reported significant reductions in the percentage change in BOLD signal intensity and the number of activated voxels in the visual cortex during lens-induced myopia^11^. This finding is consistent with the results of our study. One study revealed that the FC between the visual cortex and various brain regions, including the left anterior cingulate gyrus, left superior parietal gyrus, left calcarine cortex and right lingual gyrus, was significantly disturbed in patients with high myopia^10^. Here, we observed that the visual cortex of myopic guinea pigs had abnormal FC not only with the left visual cortex but also with the hypothalamus, entorhinal cortex, and periolfactory cortex. Compared with previous findings^10^, in addition to abnormal visual cortex FC, differences in other cortical regions may be due to differences in species, and the myopia model constructed in this study reached only moderate rather than high myopia. ReHo-Cerebral blood flow (CBF) changes in the orbital part of the inferior frontal gyrus are positively correlated with best-corrected visual acuity and refractive diopter changes in patients with high myopia^50^, while average ALFF signal values in different regions are not correlated with myopic performance^51^, and ALFF values in the cerebral area of the left parahippocampal gyrus, cerebellar vermis and left posterior cingulate cortex were significantly increased in the same patient after Lasik surgery^52^. However, the difference in ALFF levels between normal and myopic brain regions has not yet been investigated. In this study, we found that the AFLL values were significantly reduced in the visual cortex of myopic guinea pigs, indicating a decrease in the excitability of neurons in the visual cortex.

The optic tract is a segment of the nerve bundle that rearranges the position of the optic nerve fibers after they have passed through the optic chiasm, and the optic chiasm further differentiates two bundles that wrap around the cerebral peduncle to the LGN. The optic nerve, optic chiasm and optic tract are all made up of the axons of RGCs. Our study revealed reduced FA, decreased AD of the optic tract and diminished optic nerve function. Neurons in the LGN receive input directly from the retina, and recent studies have demonstrated that retinal input is required for the functional integration of dorsal lateral geniculate nucleus (dLGN) interneurons, thereby controlling the excitability of the ‘thalamic–cortex’ circuit^53^. Although a previous study of the LGN in visual disorders revealed that the response selectivity of the LGN of the amblyopic eye is reduced and that the effective connection to V1 is weak^54^, the lack of retinal input significantly affects the migration of LGN interneurons outside of bilateral optic nerve amputation^53^. In addition, we also found that knocking down retinal FOS expression in myopia can reduce damage to the visual path, that is, inhibit TSD. Our results provide more specific mechanisms for anterograde TSD in myopia, such as pathological changes in the optic tract, optic chiasm and LGN, which lie between the RGCs and the visual cortex.

On the basis of the above findings, our study not only confirms the existence of anterograde trans-synaptic degeneration in myopia for the first time but also suggests that RGC apoptosis causes structural damage to the optic tract, optic chiasma and LGN neurons, and functional damage to the visual cortex, resulting in abnormal visual information transduction pathways. Knocking down FOS expression inhibited RGC apoptosis, inhibited damage to downstream pathways and slowed the progression of myopia. These findings provide a new experimental basis for understanding the pathogenesis of myopia and offer a new method for precisely targeting the quality of myopia.

### Reduced excitability of neurons in the visual cortex and exacerbated synaptic remodeling in myopia

We found that the number of excitatory neurons in the myopic visual cortex decreased, the neuronal structure was damaged and Ca^2+^ efflux and ALFF values decreased, indicating the inhibited excitability of neurons in the myopic visual cortex. In previous studies of visual abnormalities, Wiesel and Hubel^55^ reported that form deprivation leads to irreversible weakening of closed-eye visual function and changes in primary visual cortex tissue. Visually deprived eyes lose much of their ability to activate the visual cortex, resulting in a reduction in the number of neurons that respond preferentially to stimuli in the eye^55,56^. In strabismic animals, excitatory intrinsic connections between adjacent OD columns in the visual cortex are selectively lost, leaving only inhibitory processes in most cells^57^. During the critical period, monocular deprivation causes the retraction of the thalamocortical afferents serving the deprived eye and the dendritic arbors of horizontal connections^58^. As both thalamocortical afferents and long-range horizontal connections are central to classical Radio frequency (RF) formation, their alteration results in reduced excitation^59^. Ca^2+^ efflux indicates excitatory inhibition in the cerebral cortex^60^, and a decrease in the ALFF also represents reduced cortical excitability.

Synapses are important structures for transmitting information between neurons, and abnormal changes in their structure inevitably lead to changes in the functioning of the nervous system. Synaptic plasticity refers to the ability of nerve cells of the brain to form new connections or to make permanent changes in the strength of existing connections^61^. Our study revealed that the expression of synaptic-related factors in the visual cortex decreased and that the synaptic structure was abnormal. We further investigated synaptic plasticity in the visual cortex, and the detection of sEPSCs and sIPSCs revealed a decrease in excitatory transmission and an increase in inhibitory transmission before synaptic stimulation. The occurrence of confirmed long-term depression (LTD) and LTP is a manifestation of the high plasticity of synapses. LTD refers to the significant and sustained decrease in the synaptic transmission efficiency of neurons after repeated stimulation^12^.

The impact of visual deprivation on the nervous system involves long-term changes in synaptic transmission efficiency, typically manifested as an increase in low-frequency stimulation, which implies a gradual weakening of interactions between neurons and a decrease in synaptic strength. This process is achieved by altering neuronal membrane potentials, presynaptic and postsynaptic neurotransmitter release, and the strength of synaptic connections. Through such adaptive adjustments, the nervous system can effectively reduce overexcitability, prevent neural activity disorders and thereby induce LTD in neural circuits^62^. Visual deprivation leads to significant changes in synaptic activity between neurons, and the excitability of retinal or visual cortical neurons usually decreases^63^. Research by Bochner et al. has shown that in amblyopic states, the visual cortex exhibits synaptic transmission inhibition and structural abnormalities, leading to a decrease in visual information processing function^64^. This inhibition directly affects the activity of neural circuits, which in turn triggers the occurrence of LTD. Specifically, the lack of visual input from the deprived eye causes adaptive adjustments in the neural circuits of the visual cortex, manifested as weakened synaptic activity, and the inhibition of thalamic input is a key factor in this process, leading to a decrease in synaptic transmission efficiency and further manifested as LTD^65^. LTD is the main cause of the loss of visual cortical responsiveness in monocular FDM, reflecting the adaptive response of the nervous system to the lack of visual input. It is specifically manifested as a decrease in synaptic transmission efficiency and inhibition of neuronal activity, leading to a loss of responsiveness of the visual system to input^66^. In addition, in FDM, the induction of LTD by electrical stimulation of the dLGN can lead to a decrease in the amplitude of visual evoked potentials in myopia models, indicating weakened neural activity in the visual cortex and further reflecting the loss of responsiveness of visual cortical neurons^67^. Mitochondria are closely related to synaptic function and their content may reflect synaptic plasticity. Studies have shown that the mitochondrial area and synaptic density in the visual cortex of FDM rats are reduced^68^. In our myopia model, the results are consistent with the aforementioned studies, indicating that myopia induction alone can lead to LTD. We agree that visual deprivation, independent of TSD, can also trigger LTD, which is an important adaptive response mechanism of the retina and brain. In the AAV-treated animal model, although RGC apoptosis may be alleviated, visual deprivation can still cause changes in synaptic plasticity in the retina and visual cortex by altering the lack of visual input. Therefore, even in the absence of RGC apoptosis, visual deprivation will still trigger LTD.

Our study also found that myopia is accompanied by apoptosis of cells such as RGCs, indicating that RGC apoptosis causes structural and functional damage to downstream neurons, suggesting the presence of TSD in myopia^69^. Studies have shown that even after only 3 days of monocular deprivation, significant structural changes occur in thalamocortical synapses, ultimately leading to large-scale axonal atrophy^70^. LTD is associated with synaptic inhibition and dendritic atrophy and loss^71^. One study revealed that autophagy in dendrites leads to the degradation of synaptic components, promoting LTD^72^. Chronic in vivo cocaine administration was found to cause a postsynaptically mediated decrease in synaptic density in medium spiny neurons in the NAc shell with simultaneous detection of LTD^73^. These studies indicate that TSD during periods of deprivation can also contribute to the occurrence of LTD.

Unlike visual deprivation or amblyopia, TSD typically occurs due to synaptic disconnection or functional loss in specific area of the nervous system. In the visual system, TSD is often triggered by damage or the loss of key structures, such as the retina or dLGN. This damage can lead to the degeneration of related neurons in the visual cortex^74,75^. TSD not only affects the strength of synaptic transmission but may also cause neuronal death and damage to synaptic connections^76^. LTD induced by TSD is generally associated with structural damage and degeneration, distinguishing it from LTD caused by visual deprivation or amblyopia. TSD is more likely to cause structural degeneration of neurons, potentially leading to damage in these neural circuits. In contrast, LTD due to visual deprivation or amblyopia is mostly a functional change in synapses, characterized by decreased efficiency of synaptic transmission, reduced synaptic activity and diminished neuronal responsiveness^77^. Overall, visual deprivation and TSD influence LTD through distinct mechanisms, operating independently of each other.

In this study, we found that experimental myopia is accompanied by apoptosis of RGCs, which causes significant dysfunction in the visual cortex of the brain, thereby inducing myopia-related anterograde TSD and triggering the occurrence of LTD. Furthermore, our research results also indicate that when AAV knockdown of FOS is used to inhibit retinal apoptosis and alleviate TSD during myopia induction, the degree of LTD is inhibited to a certain extent compared with the myopia group alone. Our results further illustrate that TSD is related to TLD in myopia and is an important factor promoting myopia development. However, this study has not yet proven that TSD is the sole factor in the occurrence of myopia. Future research should explore the long-term effects of different visual deprivation and TSD models on various levels of the visual system (such as the retina and visual cortex) by optimizing experimental designs. With the aid of more molecular biology and electrophysiological techniques, we can better understand the relationships between visual deprivation, TSD and LTD.

Our findings demonstrate that the apoptosis of RGCs is accompanied by damage to the optic tract and optic chiasm, as well as structural abnormalities of the LGN, leading to significant dysfunction of the visual cortex in the brain, thereby inducing the occurrence of anterograde TSD in myopia. In addition, we provide evidence that anterograde TSD reduces the excitability of visual cortex neurons and inhibits synaptic transmission in myopia, leading to exacerbated synaptic remodeling. Furthermore, we also found that FOS knockdown can inhibit RGC apoptosis and the occurrence of anterograde TSD, thereby reducing the degree of damage to synaptic plasticity. The inhibition of synaptic transmission increases myopia development, exacerbates downstream damage and destroys synaptic plasticity. Taken together, our findings reveal a dysfunction of eye‒brain coordination in myopia, providing a new experimental basis for the pathogenesis of myopia and new insight into the targeted treatment of myopia in clinical practice.

#### Limitations

ScRNA-seq has not yet been performed after Fos intervention to observe the changes in the number of neurons after Fos intervention and in which cell types the expression levels of synapse-related factors are changed.

## Supplementary information

Supplementary Information

## Data Availability

All data supporting the findings of this study are available within the Article and its Supplementary Information.
