# Supplementary material for: Single-cell RNA sequencing reveals anterograde trans-synaptic degeneration and exacerbated synaptic remodeling in myopia
Source: Exp Mol Med. 2025 Jul 3;57(7):1536–54. doi: 10.1038/s12276-025-01489-y (PMC12322287; doi:10.1038/s12276-025-01489-y)
Supplement: Supplementary file 1 — Supplementary Information [file 12276_2025_1489_MOESM1_ESM.pdf]

Supplementary materials

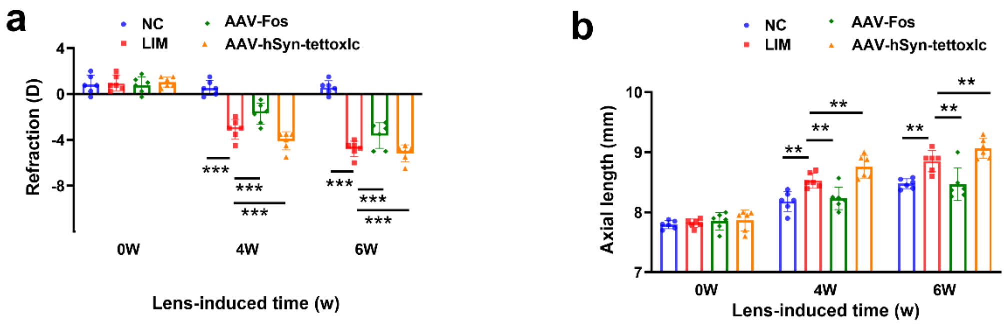

Supplementary Fig. 1 Changes in diopter and axial length in NC and LIM groups after myopia induction. a. The mean values of refraction in the right eyes of the guinea pigs after myopia induction for 0, 4, and 6 weeks between the LIM and NC groups (***P < 0.001) (n=6). b. The mean values of axial length in the right eyes of the guinea pigs after myopia induction for 0, 4, and 6 weeks between the LIM and NC groups (**P < 0.01) (n=6).

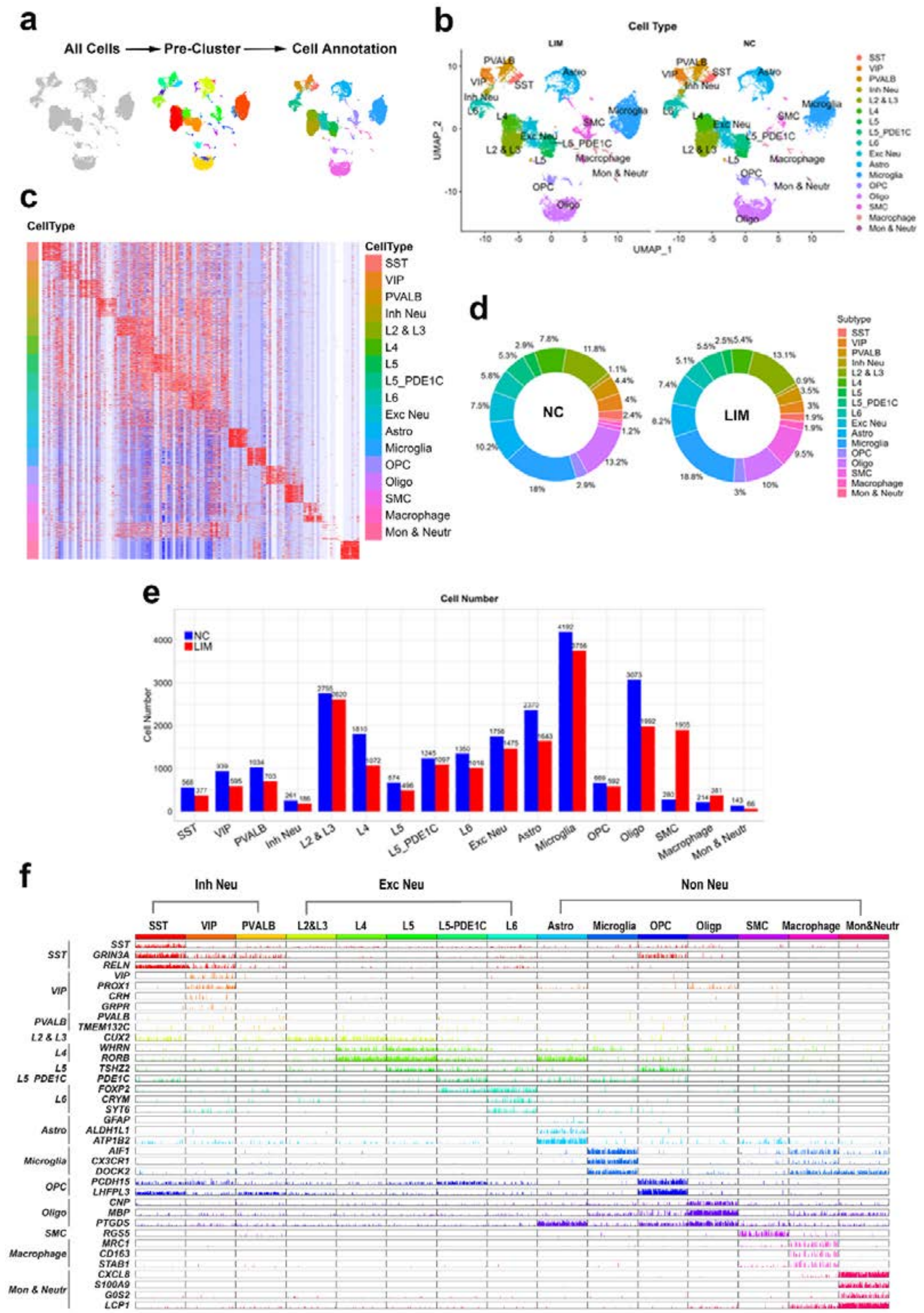

Supplementary Fig. 2 Single-cell RNA sequencing of 17 cell types of the visual cortex in experimental myopia. a. Cell clustering and annotation process. b. UMAP visualization of transcriptomic diversity in the visual cortex of 17 cell clusters in the NC and LIM groups. Cell types are shown in different colors. c.

Gene expression (rows) in individual cells (columns) arranged by cell type (left bar). d. Changes in cell proportion in 17 cell clusters in the LIM group vs. the NC group. e. Changes in the cell number in 17 cell clusters in the LIM group compared with the NC group. f. Differential gene expression confirmed the identification of cell marker genes for specified 17 cell clusters. Each cluster is represented by a color bar with the name and number of core cells representing that type.

UMAP: Uniform manifold approximation, NC: normal control, LIM: lens-induced myopia,

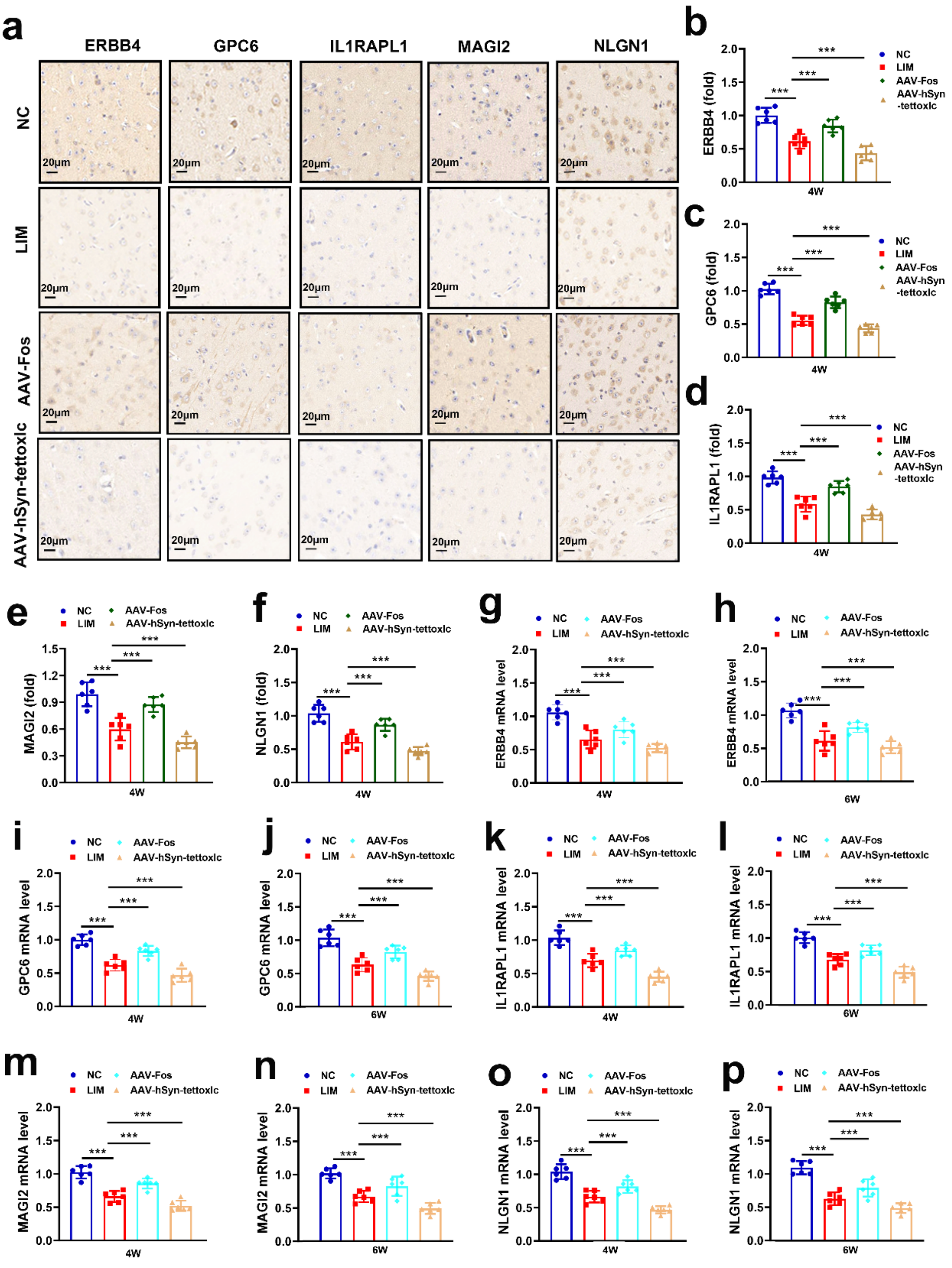

Supplementary Fig. 3 ERBB4, GPC6, IL1RAPL1, MAGI2, and NLGN1 expression at gene and protein levels detected by immunohistopathology and RT-qPCR after 4- and 6-week myopic induction in NC, LIM, AAV-Fos, and AAV-hSyn-tettoxlc groups. a. Immunohistochemical analysis of the ERBB4, GPC6,

IL1RAPL1, MAGI2, and NLGN1 proteins. b-f. Bar graphs of immunohistochemical staining for ERBB4, GPC6, IL1RAPL1, MAGI2, and NLGN1 (***P < 0.001), (n=3). g-p. Bar graphs of ERBB4, GPC6, IL1RAPL1, MAGI2, and NLGN1 expression detected by RT-qPCR (***P < 0.001) (n=6).

RT-qPCR: real-time quantitative PCR**.**
